# Structure learning enhances concept formation in synthetic Active Inference agents

**DOI:** 10.1371/journal.pone.0277199

**Published:** 2022-11-14

**Authors:** Victorita Neacsu, M. Berk Mirza, Rick A. Adams, Karl J. Friston

**Affiliations:** 1 Wellcome Centre for Human Neuroimaging, Institute of Neurology, University College London, London, United Kingdom; 2 Department of Psychology, University of Cambridge, Cambridge, United Kingdom; 3 Department of Neuroimaging, Centre for Neuroimaging Sciences, Institute of Psychiatry, Psychology and Neuroscience, King’s College London, London, United Kingdom; 4 Department of Computer Science, Centre for Medical Image Computing, University College London, London, United Kingdom; 5 Max Planck Centre for Computational Psychiatry and Ageing Research, University College London, London, United Kingdom; Technische Universitat Dresden, GERMANY

## Abstract

Humans display astonishing skill in learning about the environment in which they operate. They assimilate a rich set of affordances and interrelations among different elements in particular contexts, and form flexible abstractions (i.e., concepts) that can be generalised and leveraged with ease. To capture these abilities, we present a deep hierarchical Active Inference model of goal-directed behaviour, and the accompanying belief update schemes implied by maximising model evidence. Using simulations, we elucidate the potential mechanisms that underlie and influence concept learning in a spatial foraging task. We show that the representations formed–as a result of foraging–reflect environmental structure in a way that is enhanced and nuanced by Bayesian model reduction, a special case of structure learning that typifies learning in the absence of new evidence. Synthetic agents learn associations and form concepts about environmental context and configuration as a result of inferential, parametric learning, and structure learning processes–three processes that can produce a diversity of beliefs and belief structures. Furthermore, the ensuing representations reflect symmetries for environments with identical configurations.

## 1. Introduction

The main focus of this work is to illustrate the importance of structure learning as implemented by Bayesian model reduction. We present a computational formulation of how agents may come to form representations and concepts by foraging their environment, and how these concepts may be shaped by structure learning (i.e., Bayesian model reduction). We attempt to capture the computational mechanisms that underwrite concept formation and associated relationships (i.e., relationships between elements within contexts and relationships between contexts), as resulting from the action-perception cycles that govern agent-world interactions. In this work, agents possess and update an internal model that entertains temporally and physically structured processes when interpreting these orderly interrelationships. Whereas processes such as inference and parametric learning have been discussed extensively in the literature [1–17], little attention has been given to the type of off-line learning we define operationally as structure learning [18,19], with the implicit computational form within the Active Inference framework. This is a nascent field of inquiry raising important questions about what it means to process information off-line. In this paper, we take the first steps toward a comprehensive process theory of structure learning, grounded in a single objective function: that of maximising model evidence. In the work by Smith et al, [19], the structure learning of concepts proceeds passively (i.e., there is only an ‘observation model’). Here, we build on this work by incorporating the ‘active’ part of the perception-action cycle, making this the first attempt to connect action and perception in the context of structure learning, and thereby moving toward a more ecologically valid account of model selection. Furthermore, this work is the first—to our knowledge—to feature a comparison of information gain between online (i.e., inference, parametric learning) and off-line (structure) learning.

The capacity to mine for similarities and detect dissimilarities across sets of experienced (sensorial or autobiographical) events is a crucial facet of structured knowledge-building. This type of relational thinking is known as *concept learning*, first proposed by Bruner, Goodnow and Austin in 1956 in ‘A study of thinking’ [20]. More specifically, concepts are mental representations that allow biological agents to compare and contrast collections or sets, and their respective elements. Various researchers have since developed and expanded on concept learning. For instance, the prototype theory of concept learning suggests that biological agents possess a central example, a ‘common representation’ of a particular set, and then judge how (semantically) far (or close) new experiences are in relation to the prototype [21]. Another example is that of abstraction of rules, whereby concepts are characterised as a set of rules, and agents assess new experiences (of objects, events, etc.) based solely on their respective properties and whether they fit the definitions or not [22,23]. A further instance is that of ad hoc categories in goal-directed behaviour [24], which describes a temporary and spontaneous type of concept formation, such as ‘things to bring on a trip’. In this scenario, knowledge from different domains is combined to form a novel temporary structure, specific to the context in play.

There is an intimate relationship between context and content: if I know the context (for example, living room, beach, street, etc.) then I can call on a conditional probability distribution over the things I expect to see there (i.e., the content): sofa, TV, coffee table; sand, water, floaties; buildings, cars, traffic signs. And if I know what I am seeing (i.e., the content), then I can infer the contexts I may plausibly be in. This bidirectional relationship is implicit in our computational model: during the inferential and learning processes, both these probability distributions are optimised simultaneously. That is, in order to find out which context is in play–and to fulfil desired outcomes–agents use information acquired in previous time-steps to infer the context in which they are operating. At the same time, this context places constraints on outcomes in the future, given the actions they take (e.g., things I expect to see if I look over there).

Concept learning spans several areas of inquiry relevant to both neuropsychology and computational neuroscience: the way (biological) agents form *concepts*, how they interpret *context* and *content*, what it means to *represent* concepts and *relationships* between different elements within a context or between contexts, what *similarity* means, how humans *categorise* environments, objects, and their elements into distinct entities, what role *memory* plays, what counts as *relevant* information, and so on. A growing body of work in concept formation and structure learning employs computational frameworks, such as non-parametric Bayesian models [25–29], where generative models are equipped with an extendable space. The focus in this instance is on whether to incorporate additional components to the generative model, and at what point. For example, in [29], concept learning is presented as inferring a hidden structure, and deciding whether the current structure should be reused, or a new structure should be created. This representative example is relevant to our current model, where we disentangle three processes: inference (about latent causes), parametric learning (learning associations), and structure learning (deciding on the best generative model). Whereas non-parametric Bayesian methods furnish one way of growing models in a principled way, our work considers Bayesian model reduction, which starts with an over-complete, overly expressive model, and then removes redundant components or model features, in order to minimise complexity. We use Active Inference as the most generic formulation of Bayes-optimal behaviour that is necessary to identify the best model or structure in terms of Bayesian model evidence.

Other related approaches to concept and structure learning from the machine learning literature involve model-based clustering algorithms such as Gaussian mixture models [30] or hierarchical deep models [31]. Determining the optimal number of clusters in these approaches ranges from fitting all the models in a family and selecting the best using a Bayes information criterion [30], to augmenting deep Boltzmann machines with hierarchical Dirichlet process priors [31]. Many of these approaches, however, require large amounts of training data, unlike their human counterparts [32] and are generally difficult to evaluate in terms of Bayesian model evidence [33,34].

In light of a common framework (i.e., Active Inference), most of these constructs can be read as different manifestations (at various levels of description) of the same computational mechanisms. In what follows, we hope to show that concept learning and formation can emerge naturally from more parsimonious mechanisms: inference, parametric learning, and structure learning, whose core imperative is that of maximising model evidence or minimising (variational) free energy.

The neurobiological literature concerning concept learning is vast [35–45]. Many models focus on hippocampal-neocortical interactions, and more specifically on the interaction between the hippocampus and the prefrontal cortex (PFC), given their wide involvement in generalised knowledge-building [46–48]. In one relevant study Mack, Love, and Preston [41] address the swiftness and flexibility of incorporating new knowledge and sensory information into existing models of the world. In their study, they employ neuroimaging and a computational model called SUSTAIN [39] to ascertain the neural mechanisms underlying this aspect of concept learning (i.e., integrating new information with pre-existing concepts). Subjects viewed and categorised complex visual objects (insects) into groups by attending to either one or two features (width of legs or antennae and pincers, respectively). Although the objects presented remained constant, they belonged to different categories based on the number of features attended and their specific combinations. Subjects therefore had to integrate new and old representations of the objects in line with this foundational structure. Neuroimaging results confirmed the computational model prediction that objects encoded by similar representations should also evoke similar neural activity patterns. Further, in the hippocampus, these conceptual representations were shown to evolve and reorganise as a result of assimilating new information (in this case, new object features).

A complementary aspect of concept learning arises in the literature on problem solving and insight. Generally speaking, there are two principal kinds of problem solving: a gradual, systematic approach towards a solution; and an instant, analytically based solution in the absence of further (sensory) evidence or external information [49]. These two approaches have convenient Active Inference counterparts: gradual updates to factorised beliefs about causes, sensations, and actions, (i.e., parametric learning) and the act of selection from some model or hypothesis space (i.e., structure learning). In terms of the concept learning literature described above, these two processes reflect the difference between forming representations (i.e., Bayesian beliefs) by steady evidence accumulation and the belief updating it entails; and forming new belief structures by associating the existing elements in novel ways (i.e., applying Bayesian principles to the beliefs themselves). Finding a new or reduced model–that provides a better explanation for the data encountered–provides a plausible formulation of insight and ‘Aha!’ moments [50–55].

Insight can be described as a sudden moment of understanding, and resolution of uncertainty about a particular problem [49,56–59]. Four essential features define the experience of *insight*: the initial impasse, the restructuring of existing associations and knowledge, the ‘Aha!’ moment, and a subjectively experienced feeling of certainty [49]. This resolution of uncertainty has important consequences for how we solicit information and explore our environment: more certainty involves less exploration, or soliciting of new information. It is noteworthy that neurobiological findings in problem solving parallel observations in category learning and concept learning: as a reaction to the mental block, the PFC is thought to switch its current approach of petitioning the hippocampus for information [59,60]. In addition to this, the ventral tegmental area (VTA) is thought to encode the precision of the decision that follows the reorganisation of associations [59]. Interestingly, empirical and theoretical work in Active Inference suggests that the role of VTA is that of encoding the precision of policy selection [5,61]. Note that we associate insight with Bayesian model selection (i.e., Bayesian model reduction), and therefore resolving uncertainty about causal structure, entailed by structure of a generative model. This work focuses on two out of four features of insight: the initial trial-and-error behaviour (associated with parametric learning in Active Inference), and the reorganisation of existing current knowledge in the absence of new information (here, Bayesian model reduction), rather than the subjective experience of insight. This work implies the use of a generative model (i.e., beliefs encoding probability distributions over observed outcomes and hidden causes). As we will see below, the reorganisation of knowledge entails restructuring these beliefs as a result of Bayesian model reduction (BMR). Belief adaptation corresponds here to parametric learning, where beliefs change gradually, based on observing data. Whereas adaptation is in relation to the environment (i.e., agents ‘adapting to’ the environment as they gather sensorial information), reorganisation is in relation to the generative model per se (i.e., agents minimising complexity, maximising model evidence). In other words, adaptation is due to, and a result of moment-to-moment interactions with the environment, whereas reorganisation entails off-line (model) optimisation in the absence of evidence. This reorganisation may or may not be adaptive (to the environment), based on whether or how the environment changes.

In Active Inference, the fastest timescale entails *inferring* hidden states of the environment generating observed outcomes. At a slower timescale, it involves accumulating evidence about contingencies, to optimise *parameters* of a generative model. At the slowest timescale, *structure learning* proceeds by redistributing the products of learning to minimise model complexity, thereby underwriting a generalisation to new experiences. Physiologically, these three levels can be thought of in terms of neuronal dynamics responsible for belief updating about states of affairs in the environment. Inference corresponds to moment-to-moment belief updating. Experience-dependent learning can be associated with Hebbian plasticity as synapses accumulate contingencies [4,62]. Finally, model selection can be associated with a form of synaptic homeostasis and the removal of redundant synaptic connections [63,64]. Computationally, all these processes rest upon the imperative of minimising variational free energy (or maximising model evidence), by changing the sufficient statistics that encode Bayesian beliefs about hidden states (i.e., inference), parameters (i.e., parametric learning), and structure (i.e., model selection through reduction) of models. These optimisation processes have been formulated as various aspects of Active Inference [3,4,55], whose biological plausibility is established to a certain degree. Here, we put these three mechanisms in play together to see if we can reproduce the cardinal aspects of concept learning, context learning and representation that emerge naturally from self-evidencing [65].

Practically, we used simulations of agents situated in a novel environment. This environment comprised several rooms, two pairs of which had an identical form. Within each room, a particular location afforded a reward. The agents had two hierarchical levels of action: they could forage within each room at the lower level, or move between rooms at the higher level. In ethology, this could be construed as a simple patch foraging paradigm [66]. To begin with, agents had an imprecise representation of the possible types of rooms they would encounter, but more importantly, they did not know *a priori* which context (i.e., room) they were in, or the unique affordances of the different rooms. By simply optimising the evidence for their model of the environment, we hypothesised that the agents would come to learn and remember the number of rooms and reward locations, thereby forming a representation of their active engagement with the environment. Beliefs over hidden states, parameters, and the structure of their (actively explored) environment are encoded by–and underlie–these representations. The computational principles underlying Active Inference have been demonstrated in other contexts such as saccadic eye movements [12,15], or at a more abstract level, prosocial behaviours [67], and emotional constructs [68]. Here, we adopt a minimal model of spatial foraging and structure learning in order to clarify underlying processes and demonstrate key ideas. This is the first paper, to our knowledge, to apply the principles of structure learning (here Bayesian model selection) to spatial foraging during an active engagement with the environment.

In what follows, structure learning will refer to Bayesian model selection, and in particular, Bayesian model reduction, to find the best model of an active engagement with the environment. In virtue of the fact that these models are based upon discrete state-space models (namely, Markov decision processes), different models are distinguished by the presence or absence of a particular mapping among discrete states. In the context of the likelihood mapping, this will be between latent states of affairs in the world and observable outcomes. This means that structure learning can be cast as exploring a space of mappings among discrete states. We will demonstrate concept learning by applying parametric and structure learning to the likelihood mappings, reading ‘concepts’ as the latent causes that generate observable outcomes. Whereas parametric and structure learning are mechanistic processes, concept learning is a teleological description of what these processes look like, from a psychological or constructivist perspective.

The remainder of this paper comprises four sections. In the first, we briefly summarise Active Inference as self-evidencing and its implicit minimisation of variational and expected free energy, and we will introduce the notion of structure learning under Bayesian model reduction. We then move on to a specific description of the generative model we use to unpack these ideas and demonstrate the learning of likelihoods under structure learning and parametric learning. The third section presents a series of simulations (i.e., numerical analyses) showcasing characteristic behaviours we hoped to elicit, and their associated belief updating. Our key hypotheses were a) as agents forage their environment, they come to form representations–that is, precise (probabilistic) beliefs encoding the structure of the environment; b) structure learning in the form of Bayesian Model Reduction (BMR) assists concept formation and performance. With ongoing exposure to the environment, we hoped to see the emergence of concept learning and improved performance both as a function of gradual (parametric) learning and BMR. That is, agents will come to learn that there is a limited number of rooms, with a particular topology, find the reward more often, and gather more reward overall. The final section reviews the numerical experiments in light of existing empirical findings in neurophysiology and ethology.

## 2. A brief account of Active Inference and structure learning

In this section we summarise Active Inference, and introduce the notion of structure learning under Bayesian model reduction. The basic idea underlying Active Inference is that biological agents are inference machines that minimise (variational) free energy or, equivalently, maximise model evidence. This can also be interpreted as self-evidencing [65]; namely, minimising uncertainty about the environment [4]. Implicit in the Active Inference formulation is a (generative) model of the environment in the form of beliefs or probability distributions that encode contingencies in the world. The environment (i.e., generative process) can be thought of as being either extrapersonal [10] or the body [17], or both [9]. Briefly speaking, the agent uses sensory data to update its beliefs about latent states and the most likely policies it should pursue. This is known as *inference*. Since perception and action are optimised in tandem, Active Inference agents hold and optimise beliefs about their own behaviour. They select actions from the posterior beliefs about policies (i.e., plans), whereby a new observation is solicited, in line with the goal of fulfilling prior preferences and resolving uncertainty. The (variational) inference process in Active Inference can therefore be thought of as optimising posterior beliefs about the causes of sensorial experience for past, present, and future (hidden) states, based on observations, and depending upon the pursuit of specific policies [4]. The process known as *parametric learning* involves the optimisation of beliefs about relationships implicit in the interaction between different (latent) variables in the environment, where actions are chosen to resolve uncertainty about the parameters of a generative model. These parameters can encode beliefs (usually as concentration parameters) about likelihood (of outcomes given hidden states), transitions (among states), preferences (for outcomes), initial states and policies. In the Active Inference literature, these model parameters are usually called **A**, **B**, **C**, **D**, and **E** matrices, respectively (please see Table 1 for a glossary of terms).

**Table 1:** Glossary of terms.

Notation/Term	Meaning
$\begin{array}{l} o_{\tau }\in \{0,1\}\\ o_{\tau }\in [0,1]\\ {\overset{⌢}{o}}_{\tau }=\;\text{ln}\;o_{\tau } \end{array}$	Outcomes, their posterior expectations and logarithms
$\tilde{o}=(o_{1},\ldots o_{t})$	Sequences of outcomes until the current time point
$\begin{array}{l} s_{\tau }\in \{0,1\}\\ s_{\tau }^{\pi }\in [0,1]\\ {\overset{⌢}{s}}_{\tau }^{\pi }=\;\text{ln}\;s_{\tau }^{\pi } \end{array}$	Hidden states and their posterior expectations and logarithms, conditioned on each policy
$\tilde{s}=(s_{1},\ldots ,s_{T})$	Sequences of hidden states until the end of the current trial
$\begin{array}{c} \pi =(\pi_{1},\ldots ,\pi_{K}):\pi \in \{0,1\}\\ \pi =(\pi_{1},\ldots ,\pi_{K}):\pi \in [0,1]\\ \overset{⌢}{\pi }=\;\text{ln}\;\pi \end{array}$	Policies specifying action sequences, their posterior expectations and logarithms
*u* = *π*(*t*)	Action or control variables for each factor
*γ*, **γ** = 1/**β**	The precision (inverse temperature) of beliefs about policies and its posterior expectation
*β*	Prior expectation of temperature (inverse precision) of beliefs about policies
$\begin{array}{c} A\in [0,1]\\ \overset{⌢}{A}=\psi (a)-\psi (a_{0}) \end{array}$	Likelihood matrix mapping from hidden states to outcomes and its expected logarithm
$\begin{array}{l} a_{}^{m}\in \mathbb{R}\\ a_{}^{m}\in \mathbb{R}\\ a\prime_{}^{m}\in \mathbb{R}\\ a\prime_{}^{m}\in \mathbb{R} \end{array}$	Prior concentration parameters of the likelihood Posterior concentration parameters of likelihood Reduced posterior of the likelihood Prior concentration parameters for the reduced model (of the likelihood)
$\begin{array}{l} B_{\tau }^{\pi }=B(u=\pi (\tau ))\in [0,1]\\ {\overset{⌢}{B}}_{\tau }^{\pi }=\;\text{ln}\;B_{\tau }^{\pi } \end{array}$	Transition probability for hidden states under each action prescribed by a policy at a particular time, and their logarithms
$\begin{array}{l} C:={\overset{⌢}{B}}_{\tau }^{0}\in [0,1]\\ \overset{⌢}{C}=\;\text{ln}\;C \end{array}$	Transition probability for hidden states under a habit and their logarithm
**U**_*τ*_ = ln *P*(*o*_*τ*_)	Logarithm of prior preference or utility over outcomes
**D** ∈ [0,1]	Prior expectation of each state at the beginning of each trial
**E** ∈ [0,1]	Prior expectation of each policy at the beginning of each trial
*Q*	Approximate posterior distribution over the latent causes of the generative model–e.g. **s**, *A*, *π*
$F:F_{\pi }=F(\pi )=\sum_{\tau }F(\pi ,\tau )\in {\mathbb{R}}^{}$	Variational free energy for each policy
$G:G_{\pi }=G(\pi )=\sum_{\tau }G(\pi ,\tau )\in {\mathbb{R}}^{}$	Expected free energy for each policy
$H=-diag(\overset{⌣}{A}\cdot \overset{⌢}{A})$	The vector encoding the entropy or ambiguity over outcomes for each hidden state
$s_{t}=\sum_{\pi }\pi_{\pi }\cdot s_{t}^{\pi }$	Bayesian model average of hidden states over policies
*Cat*(*A*) *Dir*(*a*)	Categorical and Dirichlet distributions, defined in terms of their sufficient statistics (probabilities and concentration parameters)
$\sigma {\left(-G\right)}_{\pi }=\frac{\text{exp}(-G_{\pi })}{\sum_{\pi }\text{exp}(-G_{\pi })}$	Softmax function, returning a vector that can be treated as a proper probability distribution
$\begin{array}{c} \overset{⌢}{A}=E_{Q}[\;\text{ln}\;A]=\psi (a)-\psi (a_{0})\\ \overset{⌣}{A}=E_{Q}[A_{ij}]=a\times a_{0}^{-1}\\ a_{0ij}=\sum_{i}a_{ij} \end{array}$	Expected outcome probabilities for each hidden states and their expected logarithms
**Bayesian surprise**	A measure of salience based on the (Kullback-Leibler) divergence between the recognition and prior densities. It measures the information in the data that can be recognised.
**Conditional density/posterior density**	The probability distribution of causes or model parameters, given some data; i.e., a probabilistic mapping from observed data (consequences) to causes.
**(Kullback-Leibler) Divergence**	Information divergence, information gain or relative entropy is a non-commutative measure of the difference between two probability distributions.
**Empirical prior**	Priors that are induced by hierarchical models; they provide constraints on the recognition density is the usual way but depend on the data.
**Entropy**	The average surprise of outcomes sampled from a probability distribution or density. A density with low entropy means, on average, the outcome is relatively predictable (certain).
**Generative model**	A probabilistic mapping from causes to observed consequences (data). It is usually specified in terms of the likelihood of getting some data given their causes (parameters of a model) and priors on the parameters
**Gradient descent**	An optimisation scheme that finds a minimum of a function by changing its arguments in proportion to the negative of the gradient of the function at the current value.
**Precision**	The inverse variance or dispersion of a random variable. The precision matrix of several variables is also called a concentration matrix. It quantifies the degree of certainty about the variables
**Prior**	The probability distribution or density on the causes of data that encode beliefs about those causes prior to observing the data.
**Surprise**	Surprisal or self-information is the negative log-probability of an outcome. An improbable outcome is therefore surprising.
**Uncertainty**	A measure of unpredictability or expected surprise (c.f., entropy). The uncertainly about a variable is often quantified with its variance (inverse precision).

Further to minimising uncertainty about hidden states and parameters, agents also minimise uncertainty about their generative models per se, also known as *structure learning*. Generative models are essentially alternative hypotheses about the potential causes that generate the agent’s observations. With structure learning, one considers competing hypotheses about these causes. Agents can therefore minimise uncertainty about their model based on model comparison, where the winning model becomes the hypothesis under which observed outcomes are the least surprising—i.e., the most likely hypothesis (having reduced all other types of uncertainty). In order to optimise the other types of uncertainty (i.e., about latent states or parameters), agents need sensorial (or factual) information, meaning that experience is needed. However, Bayesian model selection (e.g., reduction) operates in the absence of further sensory experience, since it proceeds by best explaining the experiences accumulated up until that point in time.

Optimality—in the current Bayesian context—includes the joint principles of optimal Bayesian decision-making under uncertainty, and the principles of optimal Bayesian design. This is most clearly seen in terms of the two parts of the expected free energy objective function that we will describe in terms of intrinsic motivation (or value)—that scores the exploratory aspect of optimal behaviour—and the second part, which is the extrinsic motivation (or value) that can be read as minimising the expected loss, or maximising expected reward. Optimality entails both maximising expected (or extrinsic) reward, and minimising uncertainty (or maximising information gain). By definition and construction, our agents are optimal in this sense.

In Active Inference, extrinsic (pragmatic) value and intrinsic value (epistemic value, i.e., novelty and salience) are optimised simultaneously. This follows because policy selection is based on expected free energy, which itself implies a dual pursuit: utility maximisation and maximising information gain [55]. These complementary imperatives are combined into a single objective function (expected free energy), such that the pragmatic and epistemic imperatives contextualise each other to provide the right balance of exploitative and exploratory behaviour.

Typically, in a novel setting, the behaviour of Active Inference agents is dominated by exploration until the epistemic values of available policies fall as uncertainty is resolved, at which point extrinsic value dominates, manifesting as exploitative behaviour. The degree to which agents explore therefore depends on the precision of their prior preferences that underwrite goals. Note that because these (extrinsic and intrinsic) values are log probabilities, their combination in expected free energy corresponds to a multiplication of probabilities. This means, a policy only has intrinsic value (i.e., information-seeking value) provided it has a non-trivial extrinsic value (i.e., goal-seeking value). For an extensive account of Active Inference and associated tenets, please see: Da Costa, Parr [1], Friston, FitzGerald [3], Friston, FitzGerald [4], Friston, Parr [7], Friston, Parr [18], Smith, Schwartenbeck [19].

In what follows, we briefly review inference, learning and model selection (a.k.a. Bayesian model reduction) in terms of belief updating that minimises variational and expected free energy.

### 2.1 Inference

The first equation describes the process of *inference* as the minimisation of variational free energy–also known as an evidence bound [69]–with regards to the sufficient statistics of an approximate posterior distribution over the hidden causes *x* (representing hidden states *s*, and policies, *π*):

$$Q(x)=\text{arg}\;{\text{min}}_{Q(x)}F\approx P{\left(x|\tilde{o}\right)}_{}{{}_{}}_{}{{}_{}}_{}{{}_{}}_{}{{}_{}}_{}{{}_{}}_{}{{}_{}}_{}{{}_{}}_{}{{}_{}}_{}{{}_{}}_{}{{}_{}}_{}{{}_{}}_{}{{}_{}}_{}{{}_{}}_{}{{}_{}}_{}{{}_{}}_{}{{}_{}}_{}{{}_{}}_{}{{}_{}}_{}{{}_{}}_{}{{}_{}}_{}{{}_{}}_{}{{}_{}}_{}{\;(1)\;}_{}Variational_{}\;{}_{}free_{}\;{}_{}energy$$

$$F=E_{Q}[\;\text{ln}\;Q(x)-\;\text{ln}\;P(\tilde{o}|x)-\;\text{ln}\;P(x)]$$ (1.1)

$$=E_{Q}[\;\text{ln}\;Q(x)-\;\text{ln}\;P(x|\tilde{o})-\;\text{ln}\;P(\tilde{o})]$$ (1.2)

$$=\underset{relative\;entropy}{\underset{︸}{D_{KL}[Q(x)||P(x|\tilde{o})]}}-\underset{\text{log}\;evidence}{\underset{︸}{\;\text{ln}\;P(\tilde{o})}}$$ (1.3)

$$=\underset{complexity}{\underset{︸}{D_{KL}[Q(x)||P(x)]}}-\underset{accuracy}{\underset{︸}{E_{Q}[\;\text{ln}\;P(\tilde{o}|x)]}}$$ (1.4)

Where $\tilde{o}=(o_{1},\ldots ,o_{t})$ designates observed outcomes to the current time. This equation can be regarded as specifying the process of perception. It shows that minimising variational free energy brings the Bayesian beliefs close to the true posterior beliefs by minimising the relative entropy term (that is never less than zero). This is equivalent to forming beliefs about hidden states of affairs that provide an accurate but parsimonious, complexity minimising, explanation of observed outcomes. Complexity here is simply the difference between posterior and prior beliefs; i.e., the degree to which one ‘changes one’s mind’ when updating prior to posterior beliefs.

Action and planning are usually formulated as selecting the action from the most plausible policy that has the least expected free energy:

$$\pi^{*}=\text{arg}\;{\text{min}}_{\pi }={\sum_{\tau }G(\pi ,\tau )}_{{}_{}}{{}_{{}_{}}}_{{}_{}}{{}_{{}_{}}}_{{}_{}}{{}_{{}_{}}}_{{}_{}}{{}_{{}_{}}}_{{}_{}}{{}_{{}_{}}}_{{}_{}}{{}_{{}_{}}}_{{}_{}}{{}_{{}_{}}}_{{}_{}}{{}_{{}_{}}}_{{}_{}}{{}_{{}_{}}}_{{}_{}}{{}_{{}_{}}}_{{}_{}}{{}_{{}_{}}}_{{}_{}}{{}_{{}_{}}}_{{}_{}}{{}_{{}_{}}}_{{}_{}}{{}_{{}_{}}}_{{}_{}}{{}_{{}_{}}}_{{}_{}}{{}_{{}_{}}}_{{}_{}}{{}_{{}_{}}}_{{}_{}}{{}_{{}_{}}}_{{}_{}}{{}_{{}_{}}}_{{}_{}}{{}_{{}_{}}}_{{}_{}}{{}_{{}_{}}}_{{}_{}}{{}_{{}_{}}}_{{}_{}}{{}_{{}_{}}}_{{}_{}}{}_{{}_{}}{\;(2)\;}_{{}_{}}Expected_{{}_{}}\;free_{{}_{}}\;energy$$

$$G(\pi ,\tau )=E_{\tilde{Q}}[\;\text{ln}\;Q(A,s_{\tau }|\pi )-\;\text{ln}\;P(A,s_{\tau },o_{\tau }|\tilde{o},\pi )]$$ (2.1)

=EQ˜[lnQ(A)−lnQ(A|sτ,oτ,π)]︸(Negative)novelty+E[lnQ(oτ|π)−lnQ(oτ|sτ,π)]Q˜︸(Negative)salience−EQ˜[lnP(oτ)]︸Extrinsicvalue (2.2)

Where $\tilde{Q}=Q(o_{\tau },s_{\tau }|\pi )=P(o_{\tau }|s_{\tau })Q(s_{\tau }|\pi )$. This equation identifies the best policy and accompanying action at the next time step. Notice that this kind of planning–based upon expected free energy–involves averaging the free energy expected following a policy under the predicted outcomes. This means the expected accuracy becomes extrinsic value; namely, the extent to which outcomes conform to prior preferences. Similarly, the expected relative entropy becomes an information gain pertaining to unknown model parameters (labelled novelty) and unknown hidden states (labelled salience). These are sometimes referred to as intrinsic values.

### 2.2 Parametric learning

*Parametric learning* can be thought of as resolving uncertainty about (generative) model parameters. Active Inference agents have implicit priors (e.g., **A**) and hyper-priors (e.g., *a*) encoding beliefs about model parameters [3]. Since parametric beliefs (e.g., **A**) are represented as categorical distributions, a suitable hyper-prior encoding the mapping between relevant couplings (e.g., state-outcome) is specified in terms Dirichlet concentration parameters. Given a state (*s*), the belief about the probability of an outcome is:

P(o|s,A)=Cat(A) (3)

$$P(A|a)=Dir(a)\Rightarrow \left\{\begin{array}{l} E_{P(A|a)}\left[A_{ij}\right]=\frac{a_{ij}}{\sum_{k}a_{kj}}\\ E_{P(A|a)}\left[\;\text{ln}\;A_{ij}\right]=\psi (a_{ij})-\psi \left(\sum_{k}a_{kj}\right) \end{array}\right.$$ (4)

Where *ψ* represents the digamma (derivative of gamma) function. Agents accumulate Dirichlet parameters as they are exposed to new observations, allowing them to learn. The updates over these parameters involve accumulating the Dirichlet parameters that represent the mapping from hidden states to the observed outcome [1,3]. For example, updates to the concentration parameters of the likelihood mapping are defined as:

$$a=a+\sum_{\tau }s_{\tau }⊗o_{\tau }$$ (5)

Where *a* and **a** represent prior and posterior concentrations parameters, respectively and **s**_*τ*_ corresponds to the posterior expectations about the hidden states.

Since accumulating (Dirichlet) concentration parameters (e.g., over the likelihood) is equivalent to the type of change observed in synaptic (Hebbian) plasticity [4,62], this specific type of update can be thought of as a synaptic strengthening, every time neurons encoding states and observations (coupled by that synapse) are active simultaneously. This is a mathematical description of Hebbian or associative plasticity. Note that in this particular example, noisy mappings correspond to an imprecise likelihood mapping (e.g., making inferences under observational uncertainty, such as being in a dimly lit room).

### 2.3 Structure learning and Bayesian model reduction

The previous two subsections summarised the computational processes entailed by online and active engagement with the environment. We now turn to a different type of learning: learning the structure of a model in the absence of new (sensorial) evidence. More specifically, this work addresses the learning of the structure of the likelihood model, between periods of active engagement with the environment. We operationally define this type of learning as *structure learning*. In light of the Active Inference framework, structure learning has a specific computational form: Bayesian model (comparison and) selection [18,19]–which includes Bayesian model reduction and expansion. Bayesian model selection entails the comparison and selection of models with the greatest (model) evidence (i.e., the least free energy) [70]. In other words, *structure learning* can be thought of as a form of model selection, where agents compare and assess alternative hypotheses defined by different (prior) configurations of their generative model [18]. When synthetic agents engage in Bayesian model selection (i.e., reduction, expansion), hypotheses about the structure of the environment are being compared against a single objective function, and specific model features or mappings are removed (or retained). Since in the Active Inference framework these (e.g. likelihood) mappings implicitly encode connection strengths [4], the reorganisation may entail the removal of existent (‘synaptic’) connections, or coupling of otherwise non-existent (‘synaptic’) connections. Although the ‘capacity’ for such connections exists in the generative model itself, the connections themselves are not ‘hard-coded’. Synthetic agents may perform an exhaustive search over the hypothesis space, by considering all the associations found in the realm of possible combinatorics (of the specific elements or features involved). To illustrate, consider the following example. An agent is looking at two distinct faces for several trials, and there are two possible emotions being conveyed (happiness, sadness). The agent starts with uniform beliefs. After a few trials, the agent ‘believes’ that face 1 is ‘happy’ and face 2 is ‘sad’. If it engages Bayesian model selection with the current posterior beliefs, it can compare the current hypothesis against the hypothesis that face 1 is ‘sad’ and face 2 is ‘happy’. If (retrospectively) there is more evidence for the second hypothesis, then its associated ‘connectivity’ changes and the trials resume with this hypothesis (i.e., model) instead. This means that although the ‘capacity’ for this specific belief structure was there, there was no ‘connectivity’ between face 1 and ‘sad’ just before Bayesian model selection. In this sense, Bayesian model selection (i.e., reduction, expansion) involves reorganisation.

In this work, the focus is on a specific form of Bayesian model selection: Bayesian Model Reduction (BMR). This involves applying Bayes’ rule to full and reduced (i.e., alternative) models and evaluating the change in free energy (i.e., log Bayes factor or model evidence) for each.

Bayesian model reduction is a post-hoc optimisation, and is applied to posterior beliefs (i.e., after all the data have been ‘seen’). Essentially, BMR refines the agents’ current beliefs based on comparing alternative models (here, likelihood models) defined in terms of their priors. This comparison entails evaluating the difference in free energy between the full model (i.e., model with the original priors) and the model defined in terms of alternative priors. Applying BMR reduces model complexity by eliminating redundant parameters.

The (relative) evidence for a full and an alternative (i.e., reduced) model with priors *a*′ can be derived by applying Bayes’ rule to both models:

$$\frac{P(Α|\tilde{o},m_{alt})}{P(Α|\tilde{o},m_{full})}=\frac{P(Α|m_{alt})P(\tilde{o}|m_{full})}{P(Α|m_{full})P(\tilde{o}|m_{alt})};$$ (6.1)

$$\frac{P(\tilde{o}|m_{full})}{P(\tilde{o}|m_{alt})}=\int d\;Α\;P(Α|\tilde{o},m_{full})\frac{P(Α|m_{alt})}{P(Α|m_{full})}\approx \int d\;Α\;Q(Α)\frac{P(Α|a\prime )}{P(Α|a)}$$ (6.2)

$$=\frac{Β(a)Β(a+a\prime -a)}{Β(a)Β(a\prime )}$$ (6.3)

$$P(Α|a)=Dir(a)=Β(a)\prod_{i}{Α}_{i}^{a-1}$$ (6.4)

For a full derivation, please see [18]. In Eqs 6.3 and 6.4, B(·) denotes the multivariate beta function. The evidence ratio in Eq 6.2 may now be expressed as the change (i.e., increase or decrease) in free energy as following:

$$\Delta F=\;\text{ln}\;P(\tilde{o}|m_{full})-\;\text{ln}\;P(\tilde{o}|m_{alt})$$ (7.1)

=lnΒ(a)+lnΒ(a′)−lnΒ(a)−lnΒ(a′) (7.2)

And

a′=a+a′−a (7.3)

Where **a**′ is the reduced posterior, **a** represents the posterior concentration parameters, *a*′ represents the prior concentration parameters defining a reduced (i.e., alternative) model, and *a* represents the prior concentration parameters defining the full model. Note the simplicity of these (local) update rules and their implicit biological plausibility [18]. The equalities in Eqs 7.2 and 7.3 allow Δ*F* to be computed in a biologically plausible way that underwrites synaptic regression or pruning. In other words, the change in free energy that would have been observed under alternative hypotheses (i.e., alternative/reduced models) can be used to remove or retain certain connections depending upon whether the free energy bound on model evidence increases or decreases. This measure is therefore used to either accept or reject alternative hypotheses (as defined by their concentration parameters). Usually, redundant parameters are removed when Δ*F* ≤ −3, corresponding to a Bayes factor approximately equivalent to 0.05, meaning that the selected (reduced or alternative) model is 20 times more likely than the full model [18].

The reduced (i.e., alternative) posteriors that emerge from the equations above—if the reduced (i.e., alternative) model is accepted—are defined as following:

$$Q(Α|m_{alt})=Β{\left(a\prime \right)}^{-1}\prod_{i}{Α}_{i}^{a\prime -1}=Dir(a\prime )$$ (8)

Bayesian model reduction is an efficient (and analytic) off-the-shelf procedure that scores reduced (i.e., alternative) models—in terms of model evidence and accompanying posterior–given the priors and the posterior under a parent model. For more technical details please see [18]. The above equations (for the likelihood matrices) describe Bayesian model reduction for Dirichlet processes that are apt for this particular model. Please see Table 1 in [18] for equations corresponding to other kinds of distributions.

## 3. The generative model

This section provides a specific description of the generative model we use to unpack and demonstrate the learning of likelihoods under structure learning and parametric learning. A generative model is a joint probability distribution over observed outcomes and hidden causes. Inference corresponds to inverting a generative model (generating consequences from causes) using observed outcomes and forming posterior expectations about the hidden states (recovering causes from consequences). Here, we describe the particular generative model used to simulate behaviour in terms of moment-to-moment belief updating, slower accumulation of evidence–in the form of associative plasticity–and structure learning, in the form of Bayesian model reduction. These distinct processes are emergent aspects of minimising the variational bound on (negative log) model evidence described above. These processes have a fair degree of biological plausibility, which enables us to simulate neuronal responses and changes in synaptic efficacy during inference and learning, respectively [4,7].

The challenge when using Active Inference schemes does not lie in devising a scheme that underwrites Bayes optimal behaviour, but rather in specifying an appropriate generative model that captures the behaviour and cognition induced by the task or problem at hand. Once the generative model has been specified, model inversion (i.e., inference, learning and selection) can proceed using standard belief updating schemes (e.g., spm_MDP_VB_X.m, available in SPM12: www.fil.ion.ucl.ac.uk/spm/software/download).

The generative model we use in the following simulations is a deep or hierarchical temporal model [8,71] based on discrete states in a partially observable Markov decision process (POMDP). It comprises two Markov decision processes, where the outputs of the higher level generate the initial (hidden) states of the lower level. These types of models have been used previously to model reading and language processing (57). Here, we use it to model spatial foraging within and between different contexts.

Each level of the generative model is parametrised by a set of matrices and vectors (more generally, arrays): a likelihood matrix encoding probabilistic mappings from states to outcomes (**A**), transition probabilities among the different hidden states (**B**), prior preferences over outcomes (**C**), and finally, priors over initial states (**D**). As described above, these matrices are parametrised with Dirichlet (concentration) parameters that are accumulated with experience: the combination of a given hidden state and outcome effectively adds a concentration parameter (i.e., a count) to the appropriate element of the likelihood mapping.

**Fig 1:**
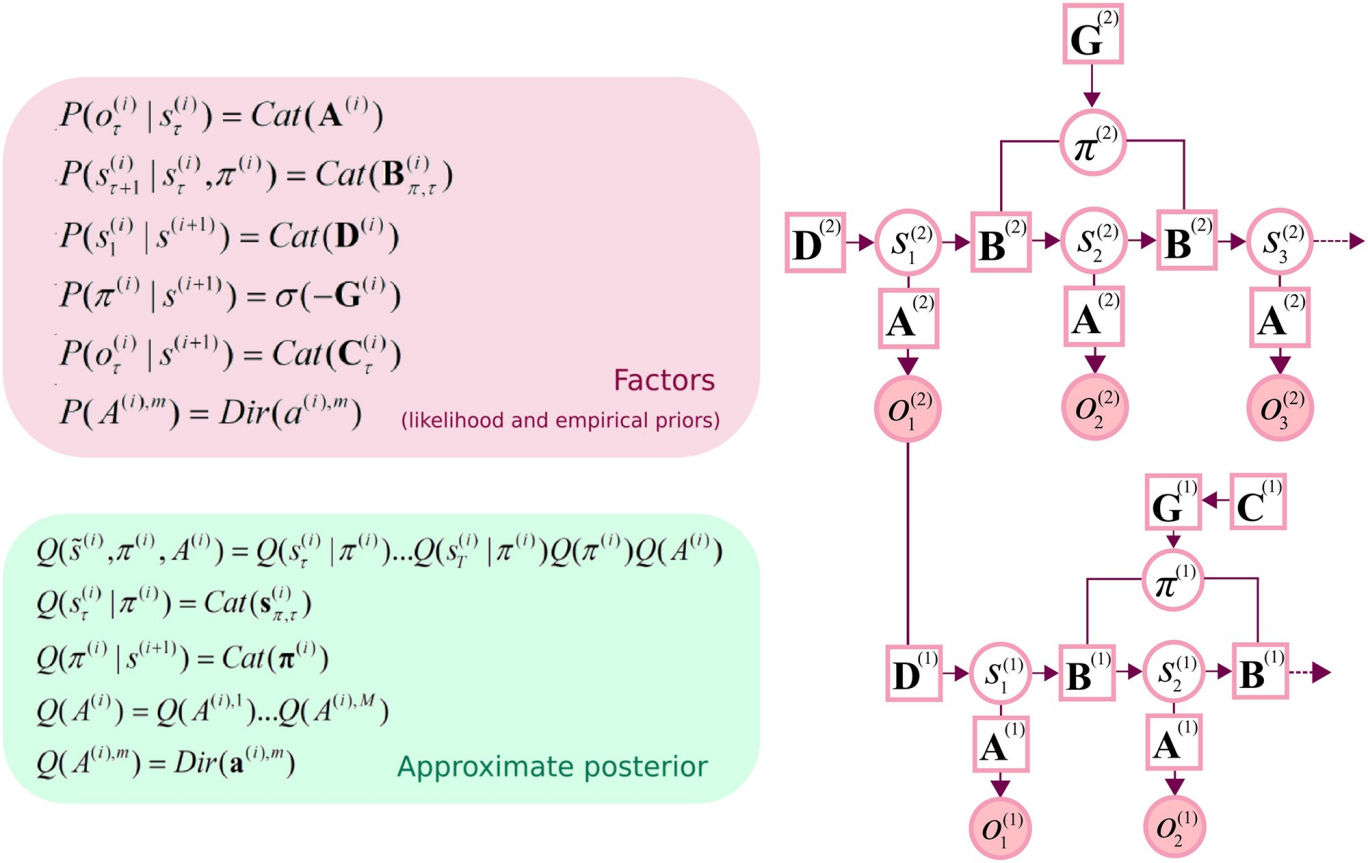
Graphical depiction of the generative model. This deep (temporal) generative model has two hierarchical levels. At the lower level there are two hidden state factors: *Location* and *context*. These generate outcomes in three outcome modalities: *Location*, *reward*, and *context* (i.e., room cue). At the higher level, there is one hidden state factor and outcome modality: *Context* (room identity); the link between the higher and lower level is via the *context* factor. Latent states at the higher level generate initial states for the lower level, which themselves unfold to generate a sequence of outcomes. Lower levels cycle for a sequence of 5 time-steps for each transition of the higher level, and there are 5 epochs in the higher level for every iteration. This scheduling endows the generative model with a deep temporal structure. The likelihood **A** is a matrix whose elements are the probability of an outcome under every combination of hidden states. **B** represents probabilistic transitions between hidden states, which depend on actions determined by policies π. **C** specifies prior preferences and **D** specifies priors over initial states. *Cat* denotes a categorical probability distribution. *Dir* denotes a Dirichlet distribution (the conjugate prior of the *Cat* distribution). Please see Table 1 for a glossary of terms.

The model used in this work generates three outcome modalities (at the lower level): the *location* within a room, a *reward* outcome, and a room or *context* specific cue (i.e., the room colour). The location modality has 16 levels corresponding to locations in a 4 x 4 grid. The reward modality has two levels: present or absent. The context modality has 16 levels corresponding to 16 possible rooms. The hidden states generating these outcomes comprise two factors: *location* (inside a specific room) and *context* (room identity). The *location* factor has 16 levels corresponding to sensed locations (i.e., 4 x 4 grid), while the *context* factor has 16 levels corresponding to the room identity (i.e., contextual cue). In other words, we have two hidden state factors (*location* and *context*) generating three outcome modalities (*location*, *reward*, and *context*) at the lower level. The link to the higher level is via the *context* hidden state factor. The content of the higher level therefore becomes the context for the lower level via this hidden state factor—please see Fig 1 for a factor graph depiction of the generative model. As an analogy, being in a specific *building* (i.e. higher level) entails a specific set of available *rooms* (lower level). For example, a school has laboratories and classrooms, each with their own configurations, types of furniture, etc. The generative model acts as a simplification of the contingencies entailed by a specific set of buildings, rooms, and their properties. Note that we deliberately reproduce the (4 x 4) structure of the lower level at the higher level (i.e., 16 rooms with 16 locations). The implication here is that the generative model can be extended hierarchically to furnish very deep inference and learning in a multiscale environment, with an implicit coarse graining over successive scales (i.e., 16 buildings with 16 rooms with 16 locations).

**Fig 2:**
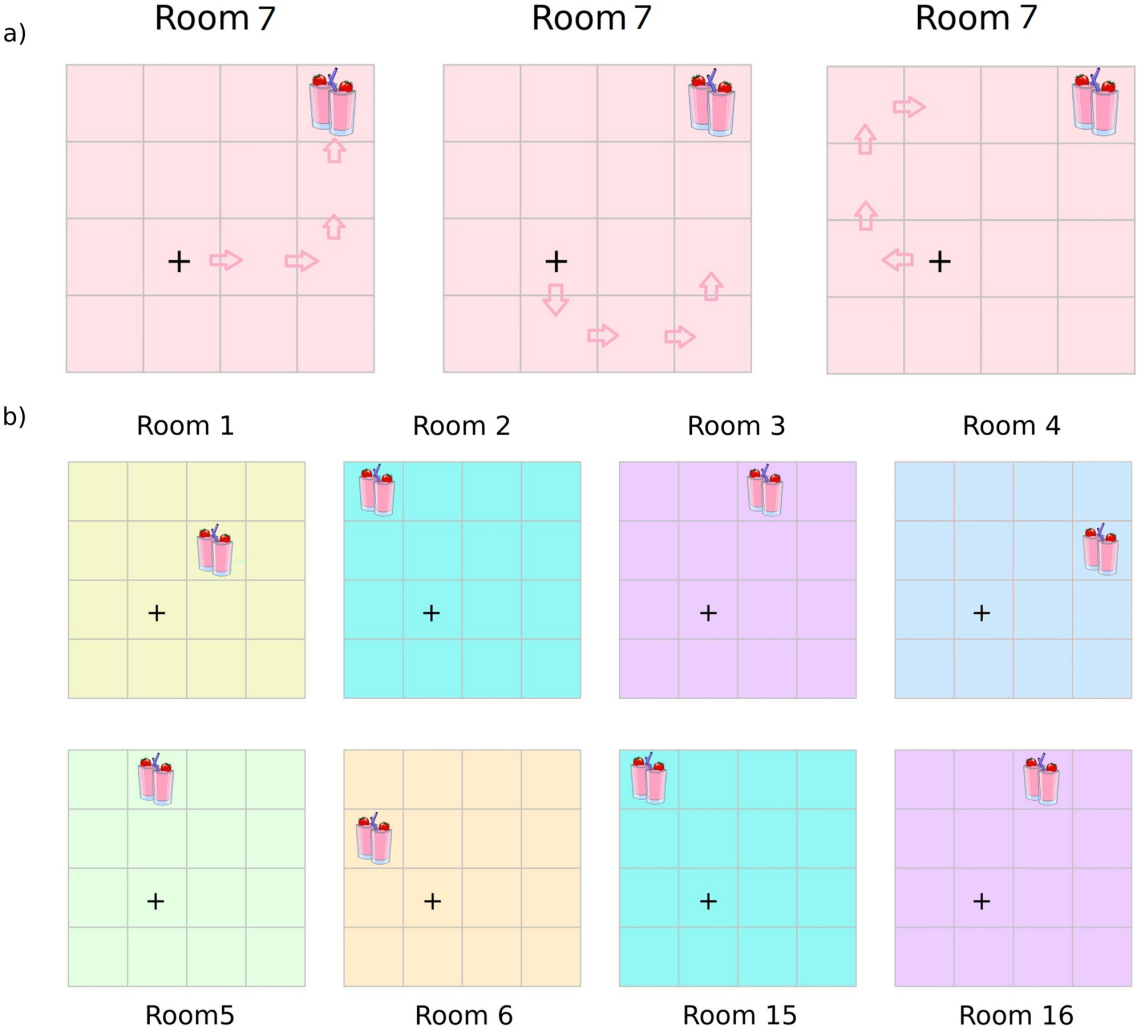
Example paths and room types. a) Examples of simulated paths or policies that agents could choose in one of the 16 possible rooms. The agents are allowed to make 4 moves, regardless of whether they find the reward or not. b) Sample rooms (out of 16 possible rooms). Rooms 2 and 15, as well as rooms 3 and 16 share the same contextual cue (colour) and reward location (locations 1 and 9 respectively). Every higher-level block involves foraging five rooms (whose identity is unknown) and exploring each of them in order for four time-steps in the lower level.

Policies entailed four moves, where each move could be in one of four directions (up, down, left, right). This means that there are 4^4^ = 256 policies (i.e., plans) that could change the location state (but not the context state). Agents could reach any of the locations in a room from the starting location within this specified number of steps given the appropriate policy (or policies). In each room, one location provided a preferred outcome or *reward*: *present* and there was a null outcome or *reward*: *absent* everywhere else. The outcomes *reward*: *present* and *reward*: *absent* were assigned a relative log probability (or utility) of 3 and 0, respectively. With these utilities, the agent would expect (or ‘prefer’) a *reward*: *present* outcome ≈ 20 times more than the *reward*: *absent* outcome. At the lower level, the starting location was always the same, namely location 7 (i.e., to the left and below the centre of the room). Please see Fig 2a for three example illustrations of simulated behaviour in one of the 16 rooms.

The structure of the second level process is similar, however, the second level *room identity* states generate the initial state of the *context* (i.e., room) factor at the lower level. More specifically, the room identity (i.e., the content from the point of view of the second level) becomes the context from the point of view of the first level.

A crucial aspect of this (deep) generative model is that the higher level generates the initial states at the lower level. This means that for every transition at the higher level, there is a succession of transitions at the lower level. For every room the agent visits, there are five time-points at the lower level. The agent had control over only the *location* state through actions at the lower level, while changes in the *context* (i.e., room) depend on the state transitions at the higher level. This diachronic construction means that the *context* state cannot change in the course of a trial at the lower level. The ensuing state transitions relax the Markovian constraints on belief updating–and accompanying behaviour–given the implicit separation of temporal scales [8]. Note that the current model features similar contexts (i.e., rooms). This is important: although agents can only forage within a specific room, they can generalise the concept of that room to other similar rooms: ‘This is a living room’ (as opposed to a bedroom). Or ‘This is an apartment’, as opposed to ‘This is an office space’.

At the lower level, agents initially have uniform beliefs about the context they find themselves in (i.e., the room identities), and they are not equipped with any preferred trajectory or sequential passage through the rooms. Furthermore, the agents have an imprecise mapping or knowledge of reward locations. This means that upon entering one of the 16 possible rooms, the agents were initially unaware of the identity of the context in which they were foraging, and believed they could be in any of the 16 rooms. Conceptually, this can be thought of as having an imprecise set of beliefs about what a room can contain: ‘I know that I am entering Room 2, but I do not know what colour or reward location it entails, nor the relationship between the room colour and reward location’.

The Dirichlet parameters encoding the confidence or precision about these various beliefs (i.e., the likelihood mapping) were set to low values, such that accumulated experience would have a substantive effect on the corresponding posterior expectations about probabilistic contingencies. Importantly, although the process generating outcomes comprises 16 rooms, there were only 14 unique rooms, as described by the contextual cue (colour) and reward location: rooms 2 and 15 are identical both in terms of the colour and reward location, and so are rooms 3 and 16 (please see Fig 2b for an illustrative sample of rooms). This means that the agents have to learn there are only 14 unique context-specific reward locations. This presents a learning problem for the agents at multiple levels. First, they have to explore each room optimally, to resolve their uncertainty about whether there is a reward or not at each location. Furthermore, they have to explore all the rooms to resolve uncertainty about which colours, and reward locations would be elicited in the different rooms they explore. The agents can therefore leverage the information they know about the rooms (i.e., configuration) in order to pursue the reward. Notice that, by construction, this hierarchical model can be extended to arbitrary depth. That is, we could have rooms of rooms of rooms, navigated at progressively slower timescales. For example, one could forage within rooms, apartments, buildings, boroughs, cities and so on, whereby agents take several steps within a room during which the apartment, building, etc., does not change.

**Fig 3:**
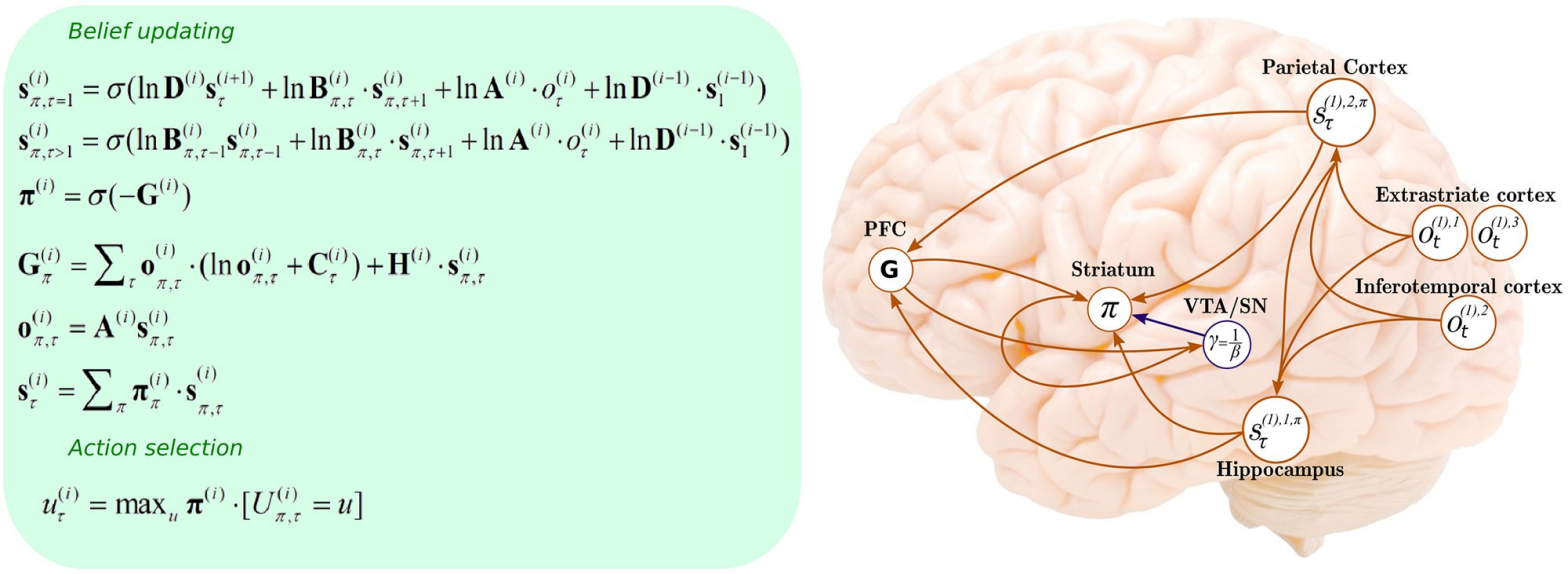
Schematic overview of belief updating. Left panel: Belief updates defining Active Inference: State-estimation, policy evaluation and action selection. These belief updates are expressed in terms of expectations, which play the role of sufficient statistics for these categorical variables. Right panel: Here, the expectations that are updated are assigned to various brain areas. This depiction is purely schematic, and its purpose is to illustrate a rudimentary functional anatomy implied by the functional form of the belief updating. Here, we have assigned observed outcomes to the occipital cortex, given its involvement in visual processing of spatial location [72,73], whereas *reward* outcomes are assigned to the inferotemporal cortex given its contributions to forming stimulus-reward associations [74]. Hidden states encoding the context have been associated with the hippocampal formation [75,76], and the remaining states encoding sampling location have been assigned to the parietal cortex, given its role in the encoding of multiple action-based spatial representations [77–79]. The evaluation of policies, in terms of their expected free energy, has been placed in the ventral prefrontal cortex. Expectations about policies *per se* and the precision of these beliefs have been associated with striatal and ventral tegmental areas, respectively, to indicate a putative role for dopamine in encoding precision [4]. The arrows denote message passing among the sufficient statistics of each factor or marginal. First and second digits in the superscript (e.g., *o*^(1),1^) indicate the hierarchical level and modality, respectively. Please see glossary in Table 1 and [4] for a detailed explanation of the equations and notation.

The generative model generates outcomes by first evaluating the expected free energy for each policy (at the higher level), and selecting the most likely policy (Figs 1 & 3). Latent states are generated based on the transition probabilities specified for this policy. Latent states then generate outcomes in one modality (for this model, *context*), and the process repeats for the lower level, whereby the outcomes are generated in three modalities: *location*, *reward*, and *context*. Perception (i.e., inference about latent states) is equivalent to inversion of this generative model (given a sequence of outcomes). Learning corresponds to parametric updates. Fig 3 summarises the associated belief updates about hidden states, policies and ensuing action selection using the free energy minimising solutions of the previous section.

This model entails online planning–in the sense that, at each point in time, the agent evaluates future trajectories in terms of expected free energy, and action is sampled from beliefs about those policies. Briefly speaking, agents form expectations about future states by projecting their posterior beliefs to the future epochs, under each policy [3]. Policies are then evaluated under these beliefs in terms of their expected free energy, which involves goal-fulfilling and uncertainty-resolving components: c.f., expected value and information gain, respectively. This renders policy selection (implicitly) contingent upon expectations of future states under each policy. It is this aspect that lends synthetic agents the ability to plan (and explore). The sampled action is more likely to originate from the policy with a lower expected free energy. The selected action generates a new observation, and the perception-action cycle continues.

Please note that for the lower level in these simulations, the entire set of policies (4^4^ = 256) is in play. The set comprises a combination of every possible move (i.e., up, down, left, right) over 4 time-points (also called deep policies). However, in a given trial, agents can eliminate unlikely policies based on their evidence using an Occam’s window (i.e., if the difference in log probability between a policy and the most likely policy is smaller than -3). This means that the agent computes combinatorics over actions up until the very last time point. The policies at the higher level refer to the potential rooms the agent can visit (i.e., 1–16); here, agents consider policies over just one time step into the future.

## 4. Simulations and results

The main focus of this work is to illustrate the importance of structure learning as implemented by Bayesian model reduction. We will demonstrate this in the context of Active Inference by showing how it enables agents to form concepts and improve their performance, as scored by information gain, and the total reward gathered.

The kind of behaviour we hoped to elicit with this generative model can be described as follows: on repeated exposure to the rooms, the agents would explore optimally, defined by a trajectory that avoids previously visited (i.e., uninformative) locations, thereby enabling the agents to learn efficiently and remember which locations are rewarding and which locations are not. Note that this learning is context-specific, in virtue of including a context factor in the generative model. That is, each room has a location with reward and contextual cue (i.e., colour) to which the agent has access, regardless of the sampled location. Preferred outcomes are time sensitive, in that the agents are only permitted to explore for up to 4 steps in each room they forage. If the reward is not found within those 4 steps, foraging in that particular room ceases, and they move to a different room. This process repeats for a given number of blocks at the higher level, namely 2, 10, 20, 30, 40, and 50 blocks (of 5 rooms each). For the lower-level process, this means that rooms were respectively sampled for 10, 50, 100, 150, 200, and 250 trials, with five time-steps each.

**Fig 4:**
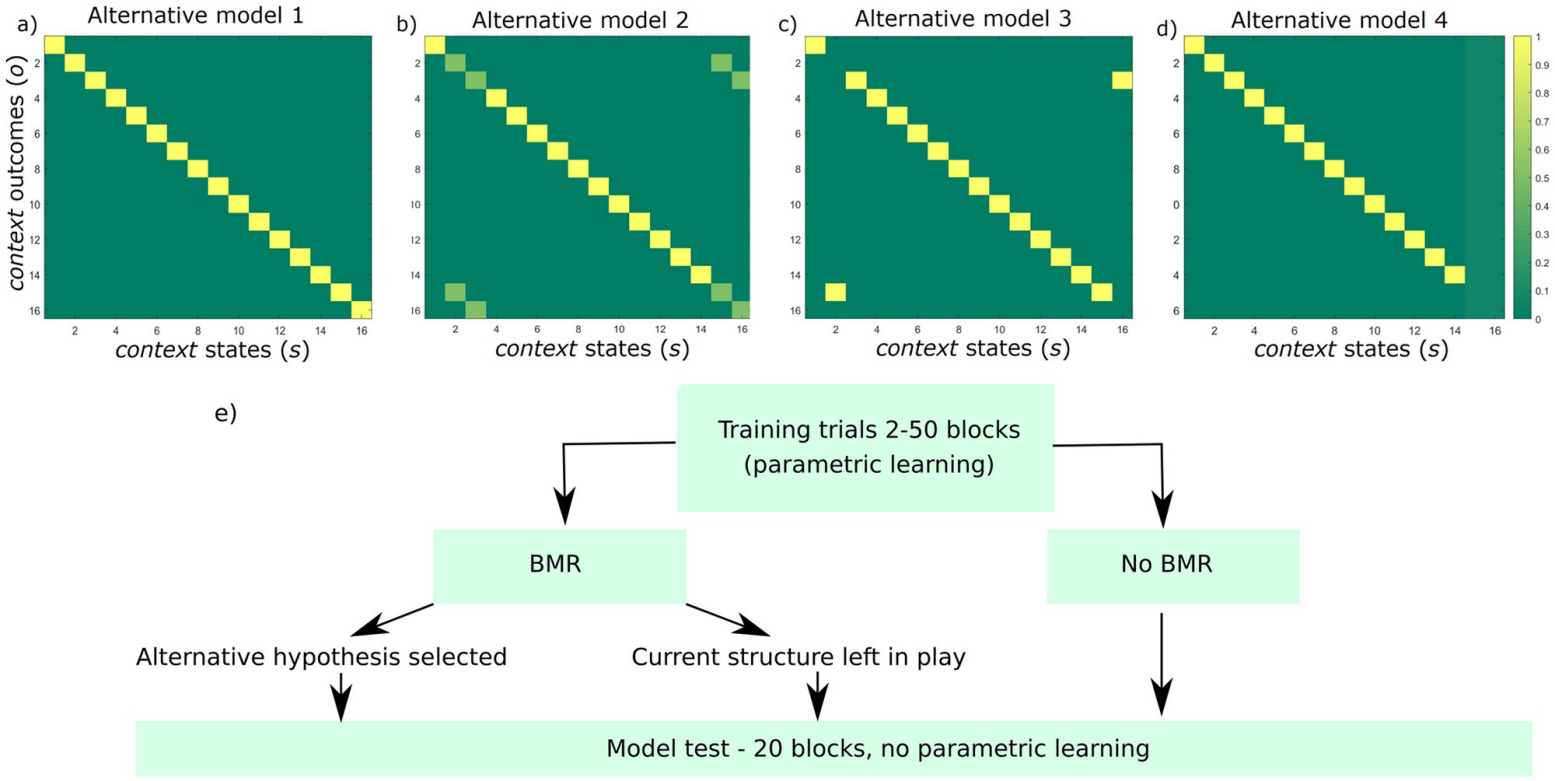
Example alternative models and flow chart depicting simulations. a)-d) Example alternative models (i.e., hypotheses). The generative process (also Alternative model 1) and alternative hypotheses were subject to Bayesian Model Reduction, focusing on the likelihood mappings encoding the *context* modality. Matrices represent a mapping from *context* states (columns) to the *context* outcomes (rows)–this can be thought of as *room identity* (*s*) to *room colour* (*o*). a) Note the identity matrix defined in the generative process is also used as an alternative hypothesis for model comparison. b) The second hypothesis depicts the identical pairs of rooms as having a 50% probability. c) The third hypothesis represents rooms 2 & 15 as being Room 15 and rooms 3 & 16 as Room 16. d) In the fourth hypothesis, rooms 15 and 16 do not exist, having a uniform distribution over all the other potential rooms–that is, rooms 15 and 16 are equally likely to have any other possible identities. e) Flow chart depicting the core simulations for all 120 agents– 60 undergoing the ‘BMR’ condition, and 60 in the ‘No BMR’ condition.

In one condition, Bayesian model reduction was employed to reassign Dirichlet parameters after each set of training blocks. This can be thought of as optimising the model structure in the absence of any further sensory information. In this instance, Bayesian model reduction is used to assess the evidence for reduced models that describe the structure of the environment. For example, one hypothesis describes an environment where each possible room has its own unique identity (despite the contextual cues and reward locations–see Fig 4a). A second example hypothesis depicts the identical pairs of rooms as having a 50% probability of mapping on to identical contextual cues (Fig 4b). Another hypothesis expresses the rooms with similar reward locations and contextual cues as sharing a representation, therefore specifying the existence of only 14 rooms (Fig 4c). In a fourth example, rooms 15 and 16 have a uniform distribution over all the potential rooms, that is, these rooms are equally likely to have any of the other possible identities (Fig 4d). These exemplar hypotheses describe potential model spaces depicting likelihood mappings for the *context* factor. Values along a column must add to 1 since they represent a probability distribution across the different possible rooms–that is, each column represents the *context* states (i.e., room identity), and each row represents *context* outcomes (i.e., room colour). Since the combinatorics of potential model spaces are extremely high, we restrained the number of potential alternative hypotheses such that they reflect concentration parameters for the likelihood *context* matrix that were observed as a result of the training trials (i.e., such that they reflect the learnt state-outcome associations).

In the first set of simulations, the model accumulated concentration parameters to learn the mappings from *context* and *location* states to the *reward* and *context* outcomes. If the free energy (i.e., negative model evidence) is lower for any of the potential hypotheses, the mixture of Dirichlet parameters is accepted and redundant synaptic connections are effectively pruned. Conversely, if the free energy is higher, the original structure is left in play. For the testing phase, we ran simulations in both conditions (i.e., models that did and did not undergo Bayesian model reduction) for a further 20 blocks at the higher level (i.e., 100 more trials involving the 16 potential rooms–please see Fig 4e for a graphical illustration of the simulation setup). During these 20 test blocks, agents in both conditions (i.e., BMR versus No BMR) were precluded from accruing further concentration parameters, such that the performance with those specific posterior distributions accumulated up to that point could be assessed (Fig 4e).

In what follows, we first show how agents learn associations and form concepts about the identities and configurations of the rooms as a result of inference, learning, and model selection (i.e., Bayesian model reduction). Next, we show the performance benefits of Bayesian model reduction, in terms of information gain, and the amount of reward accumulated. The next set of simulation results addresses the capacity of agents to learn and infer that certain rooms are identical, as defined by reward location and contextual cue (i.e., colour). Finally, we show one source of individual differences in concept formation, namely how a stronger preference for obtaining reward impacts concept acquisition and performance.

### 4.1 Agents form concepts by inferring and learning the structure of their environment

For the training section of these simulations, agents started with uniform beliefs about which context (i.e., room) they find themselves in. As far as they were concerned, upon entering a (randomly) chosen room, they could be in any of the 16 possible room types. This is specified in the structural prior of the Dirichlet concentration parameters of the *context* likelihood matrix, initialised as a uniform 10^−1^. The *reward* concentration parameters were initialised as 10^−1^ (i.e., imprecise priors) for the most plausible associations, and 0 otherwise. This means that agents had a set of initial beliefs about the configuration of rooms (in terms of potential reward locations), but were unable to make use of them without being able to discern the identity of the rooms. Initial concentration parameters over these modalities can be thought of as an *a priori* set of associations (i.e., synaptic connectivity) about contingencies in the world that sets the scene for subsequent inference and learning. Initialising the *context* likelihood mapping with uniform concentration parameters—and the *reward* likelihood mapping with small concentration parameters for the most plausible associations—is based on the following considerations: initialising with uniform concentration parameters would cause the agent to attribute any context and reward outcomes equally to all room identities (i.e., a uniform posterior distribution over the room identity states), essentially preventing the agent from learning distinct associations between states and outcomes. We could have initialised likelihood mappings with random concentration parameters, but this would have destroyed the relationship between true and learnt room labels (e.g., room 1 is learned as room 5). For a straightforward interpretation of the reduced models (used in the BMR analysis), we therefore used the reward modality to resolve ambiguity about room identity.

**Fig 5:**
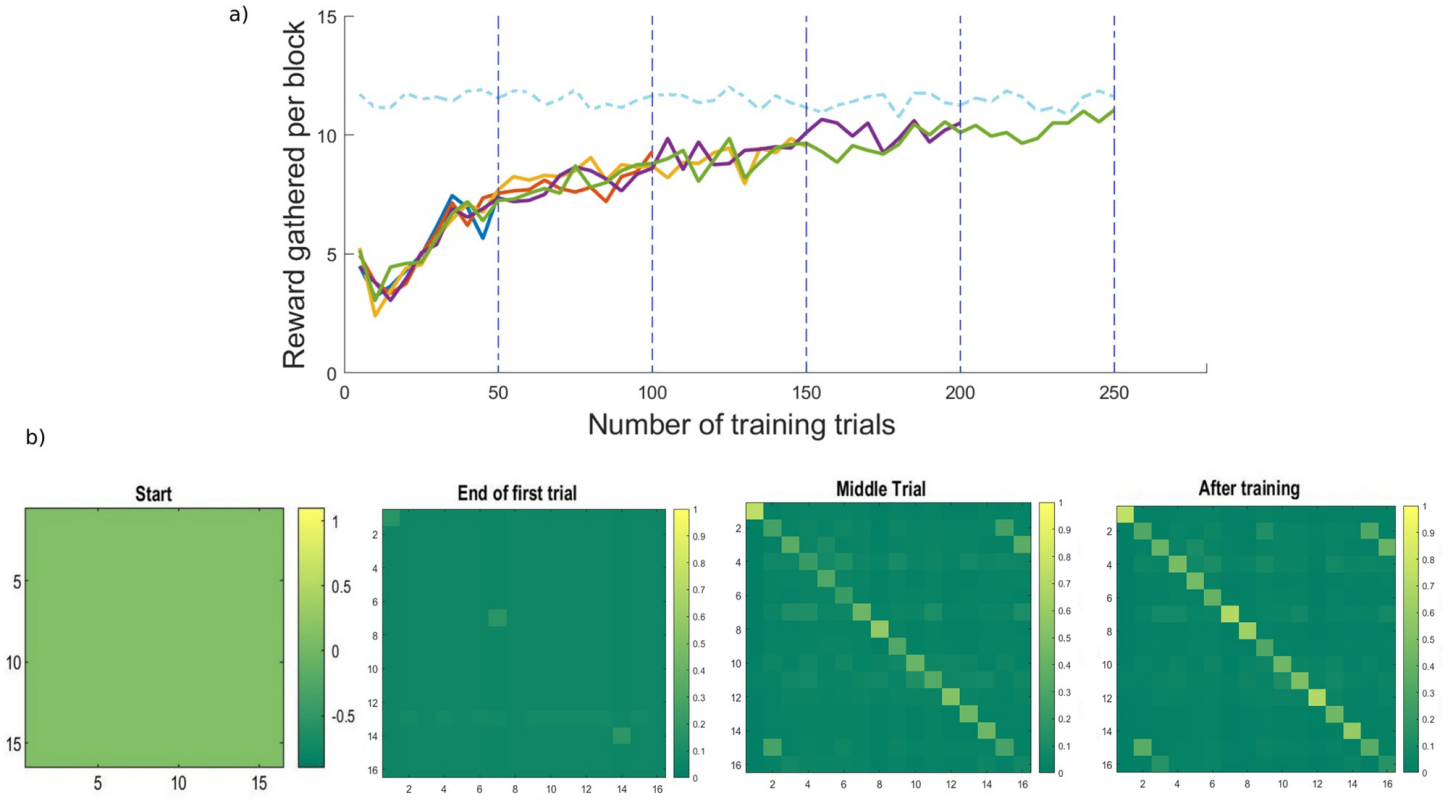
Average performance with learning. a) Progressive increase in performance scored by the amount of reward gained per block. For each higher-level block, five rooms at the lower level are explored. The performance is averaged over 20 simulated agents for each of the training settings: 10, 20, 30, 40, and 50 blocks (i.e., 50, 100, 150, 200, and 250 rooms respectively). Please note that the dashed blue line illustrates a cap in performance, represented by total reward gathered per block, averaged over 20 fully knowledgeable agents foraging for N = 50 blocks (i.e., agents that start with fully precise likelihood matrices). As agents progress through the simulations, they accumulate more reward per trial. The concavity at the beginning of training reflects exploratory behaviour; i.e., intrinsic value predominated over the extrinsic value of rewards. b) Learning: The progressive updates to the concentration parameters over state-outcome associations from a uniform distribution to a more precise one, representing concept formation. The agent forages for N = 50 blocks at the higher level (i.e., 250 lower-level trials). Middle trial represents the end of block number 25 (at the higher level).

As trials progress, agents update their beliefs about room identity (i.e., context) reflected in both the configuration of their associations (**a** matrix), and the increased probability of finding the reward. Fig 5a shows the averaged performance for agents foraging 50, 100, 150, 200, and 250 times (i.e., 10, 20, 30, 40, and 50 blocks respectively at the higher level), in terms of reward accumulated. Performance per block increases for all the agents, with an initial concavity, suggesting a preference for exploratory behaviour. In Fig 5b we show how the beliefs about context change through time for one agent, and accordingly becoming increasingly precise. Updates to the likelihood concentration parameters proceed as described above. A simple interpretation of this (parametric) learning is a change in connectivity between observations and the specific context, quantified by the number of times they are inferred to co-occur [4]. For this model, this corresponds to the number of times the reward location and contextual cue are associated with a particular room. In Fig 5b, rows represent the *context* outcome (i.e., room colour) and, and columns represent context state (i.e., room identity). This exemplifies the notion of concept acquisition: agents start with uniform beliefs about state-outcome associations and as a result of inference and learning, they acquire an explicit (and reasonably precise) representation of environmental contingencies

The purpose of this section was not only to show that synthetic agents are able to form concepts (resulting in better performance), but also to validate the generative model in its current implementation and set the scene for illustrating benefits of BMR in later sections. Although Fig 5b depicts one particular agent’s learning trajectory as it forages its environment—there are 6 training conditions: we stop the training after 2, 10, 20, 30, 40, or 50 blocks. For each of the 6 conditions, there are 20 agents. This numerical experiment is used later to illustrate how state-outcome contingencies (and therefore performance) change with or without BMR, and whether these effects vary with the amount of training.

### 4.2 Performance benefits of employing BMR

**Fig 6:**
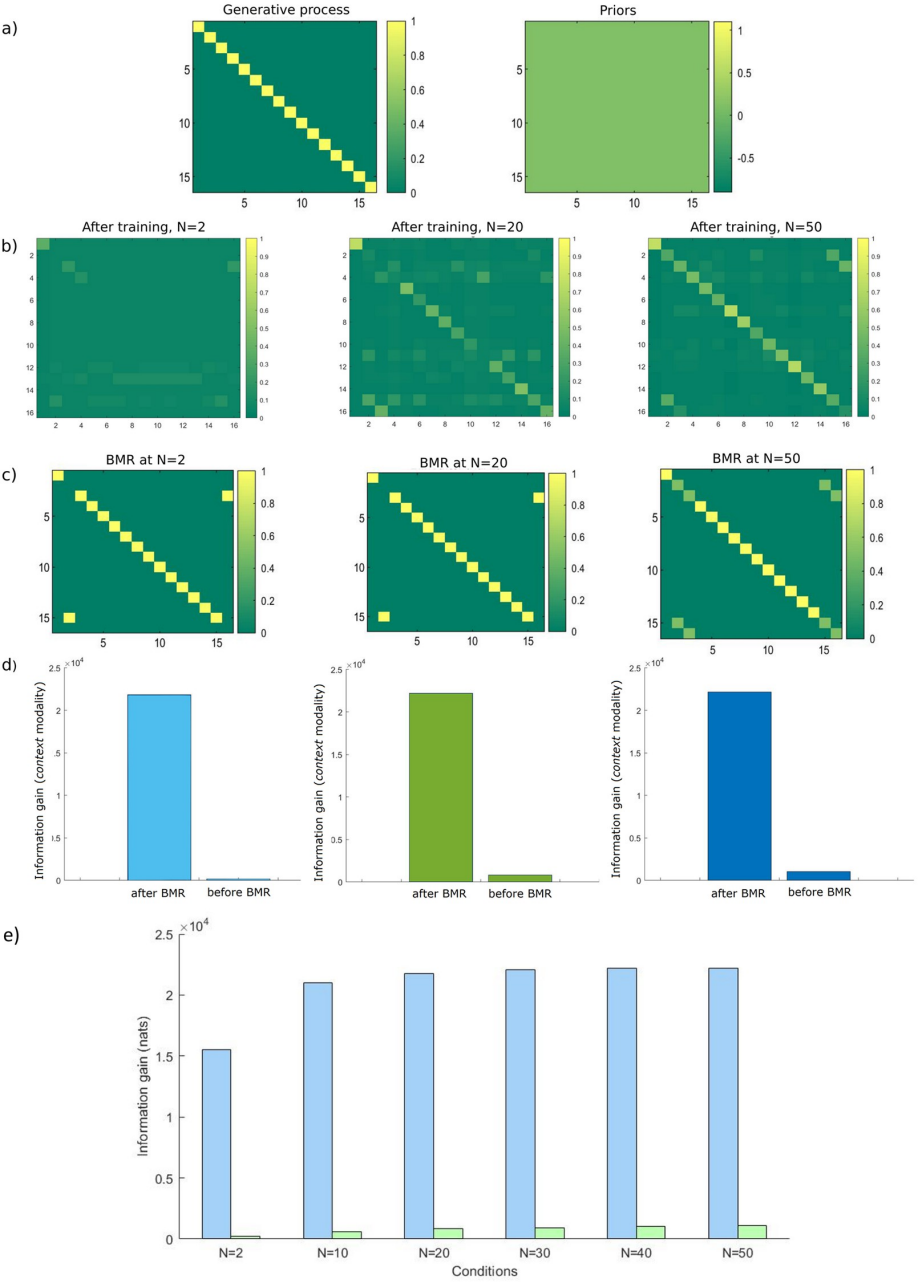
Likelihood mappings from hidden states to *context* outcomes, before and after BMR, and how these learned mappings affect concept formation for three different agents. Matrices represent the likelihood mapping from *context* states (i.e., columns) to *context* outcomes (i.e., rows). a) The process generating the actual state-outcome mappings (left) and the uniform concentration parameters that agents start with (right). b) Likelihood matrices for the three agents (averaged over all locations) at the end of 2, 20, and 50 training blocks at the higher level of foraging (from left to right). c) Likelihood matrices after BMR, showing the reduced set of state-outcome associations (i.e., likelihood) for the *context* factor. d) Information gain for the *context* modality before and after BMR for each of the three agents. e) Comparison of information gain before and after BMR, averaged over agents for each condition; light blue bars denote information gain after BMR whereas light green bars denote information gain before BMR (i.e., after N training blocks).

Along with a gradual learning of contingencies about the external world, concept formation can also be a result of, and enhanced by, Bayesian Model Reduction–a faster and saltatory type of learning. In Fig 6 we show results for three of the (120) simulated agents. We can see in Fig 6a (left panel) the generative process generating the data; that is, the ‘environment’ that agents forage. It is useful to consider here the distinction between the generative process (i.e., environment) and the generative model (i.e., the agent). Active Inference agents do not have direct access to knowledge about (hidden) states of the environment and must infer them based on observable outcomes. Although the structure of the environment that generates data is an identity mapping (mapping from hidden states to observable outcomes) in the current work, this does not preclude the agents from acquiring a different mapping; so long as it helps the agents recognise the environment in a useful way that allows them to minimise uncertainty and gather rewards. In other words, the representations (i.e., concepts) that agents form, need not be identical to the actual form of the environment (and seldom are).

As previously mentioned, all agents start their foraging with an imprecise uniform distribution over their representations (of room identities). This means that they are not aware which rooms they are foraging in (Fig 6a, right panel). Fig 6b shows the posterior concentration parameters for three different agents after training for 2 (6b, left), 20 (6b, centre), and 50 (6b, right) blocks (at the higher-level)—i.e., after 10, 100, and 250 training trials respectively). Training blocks consist of inferential and parametric learning processes described above. During this training period, therefore, agents learn gradually as they accumulate evidence about contingencies in the environment. After these training blocks, agents undergo BMR. As a result of BMR, redundant parameters are pruned, and agents form more precise representations about contingencies in the environment (encoded as state-outcome associations–Fig 6c). Because BMR maximises the evidence for a particular model or hypothesis, it does not just find the simplest possible model, but discovers the best balance between accuracy and complexity, thereby precluding over-pruning. Fig 6d quantifies the associated information gain using the Kullback-Leibler (KL) divergence between the posteriors and priors over likelihood parameters. The KL divergence is measured in nats: units of information based on natural logarithms. It can be seen in Fig 6d and 6e that Bayesian model reduction greatly enhances information gain. Furthermore, engaging BMR after 2 training trials provides a very marked change in concentration parameters relative to the two other conditions (Fig 6b and 6c). We can also see in Fig 6d that agents that experienced fewer blocks ended up selecting different alternative hypotheses as compared to the agents training for more blocks after undergoing BMR. We expand on these results in the next section.

Fig 6e shows a comparison of information gain (in nats) before and after BMR. This is averaged for all agents in each of the 6 conditions (i.e., N = 2, 10, 20, 30, 40, 50). As expected, information gain shows an upward trend with the amount of training blocks both before and after BMR. The trends observed in Fig 6d for the three different agents hold for the entire set of agents: there is a marked difference in information gain when comparing between before (light green bars) and after (light blue bars) undergoing BMR.

**Fig 7:**
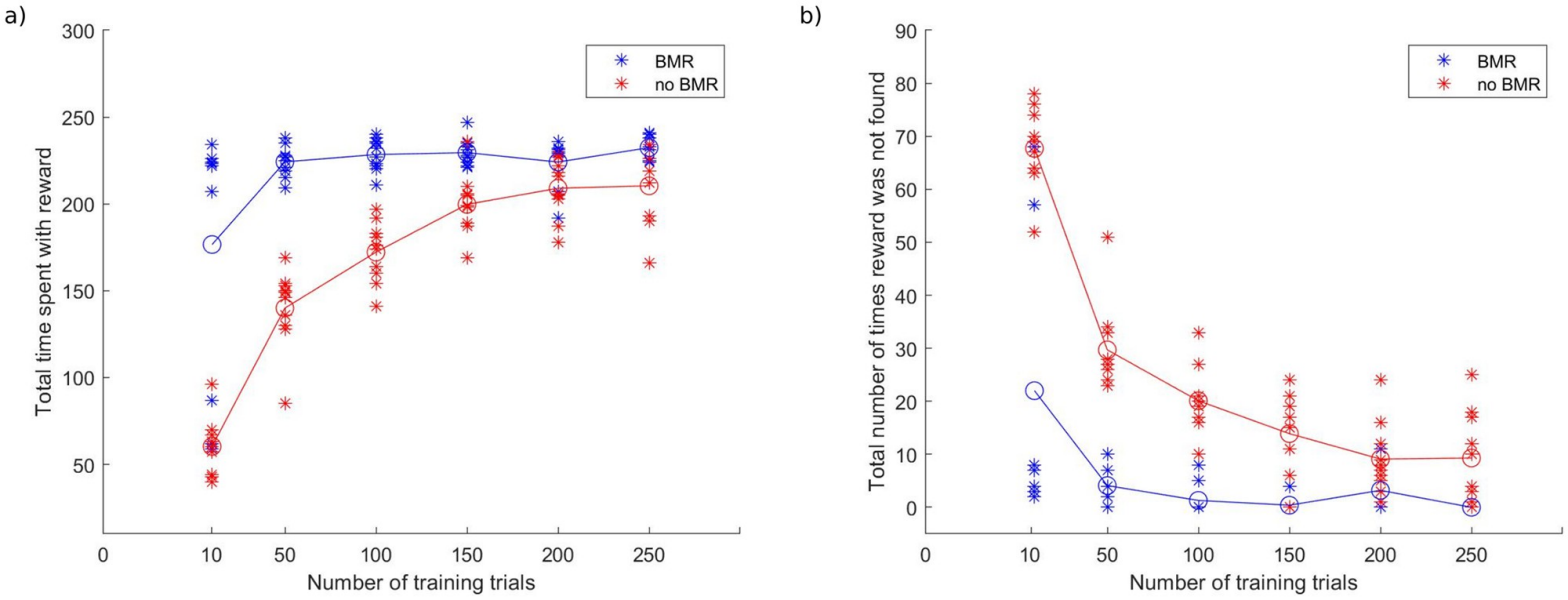
Performance comparison between agents undergoing BMR versus continuing with the posteriors accumulated after a specified number of training trials (at the lower level). Each asterisk represents an agent; circles represent performance averaged over agents at a specified number of training trials and their respective condition (BMR vs No BMR). Agents with *n* training trials are assigned to one of the two conditions, and then continue to forage for another 100 (lower level) trials. a) Total reward gained–agents undergoing BMR perform almost at peak, even before foraging through all of the 16 rooms. b) The number of times reward was (not) found–agents in the ‘no BMR’ condition spend more time foraging without finding the reward. Performance improves for agents in both conditions as they undergo more training trials.

Next, we turn to the benefits of Bayesian Model Reduction for goal-directed behaviour in terms of performance, defined as the time spent with reward–and how often the reward was found. As described in the introduction, we can regard BMR as off-line hypothesis testing in the absence of further information. Fig 7 shows the comparison between the two conditions in question: BMR versus No BMR. In a training phase, agents foraged the environment for either 10, 50, 100, 150, 200, or 250 trials (i.e., 2, 10, 20, 30, 40, and 50 blocks at the higher level). In one condition, the agents were subject to Bayesian model reduction (i.e., ‘BMR’ condition), as described above, and thereafter continued foraging the rooms for a further 20 blocks (at the higher level). In the other condition, agents continued foraging the rooms with the posteriors accumulated during the training trials, without BMR (i.e., ‘No BMR’ condition).

Choosing to stop training after different numbers of blocks (i.e., 2, 10, 20, 30, 40, 50 blocks) allows for a comparison between different stages in the learning trajectory and its effects on performance: we can see in Fig 7 that in the ‘No BMR’ condition there is a sharp jump in performance from 2 to 10 to 20 training blocks, which levels out with an increased number of training blocks, but remains below the performance for agents in the ‘BMR’ condition.

Agents in the BMR condition appear to perform almost at peak even before foraging in all of the 16 room types. This is because although the agents are not exposed to new sensory information, the beliefs encoding the *context* factor become very precise, precluding further epistemic foraging, and therefore emphasising extrinsic value (i.e., agents become relatively more exploitative). The performance for the agents in both conditions improves gradually, levelling off after training for approximately 50 higher level blocks (i.e., 250 lower-level trials). Furthermore, the agents in the ‘No BMR’ condition spend more time foraging the rooms without finding any reward, a performance characteristic that does improve with more training.

### 4.3 Agents can learn that rooms with similar configurations are identical

One aspect of concept formation concerns the ability to represent invariance and symmetries. In this section, we show that agents learn to associate the rooms with identical configurations (i.e., colour and reward location) to form associations that encode similarity, defined as the state-outcome connectivity of the *context* factor. This is a result of learning; however, this aspect is evinced more clearly following Bayesian Model Reduction. Most importantly, none of the simulated (sixty) agents undergoing BMR settled on the hypothesis specifying an identity mapping for the *context* factor. This means that despite having a process generating observed outcomes (i.e., generative process) with an identity mapping (i.e., each room has an individual identity), none of the agents judged this mapping (of state-outcome associations) as being the most parsimonious (i.e., explaining the observations accurately, in as simple a way as possible). The representations (i.e., concepts) formed by synthetic agents can–but do not have to—reflect the actual form of the environment, as long as they aid synthetic agents in interpreting the environment in a useful way (i.e., allows them to minimise uncertainty and gather rewards).

**Fig 8:**
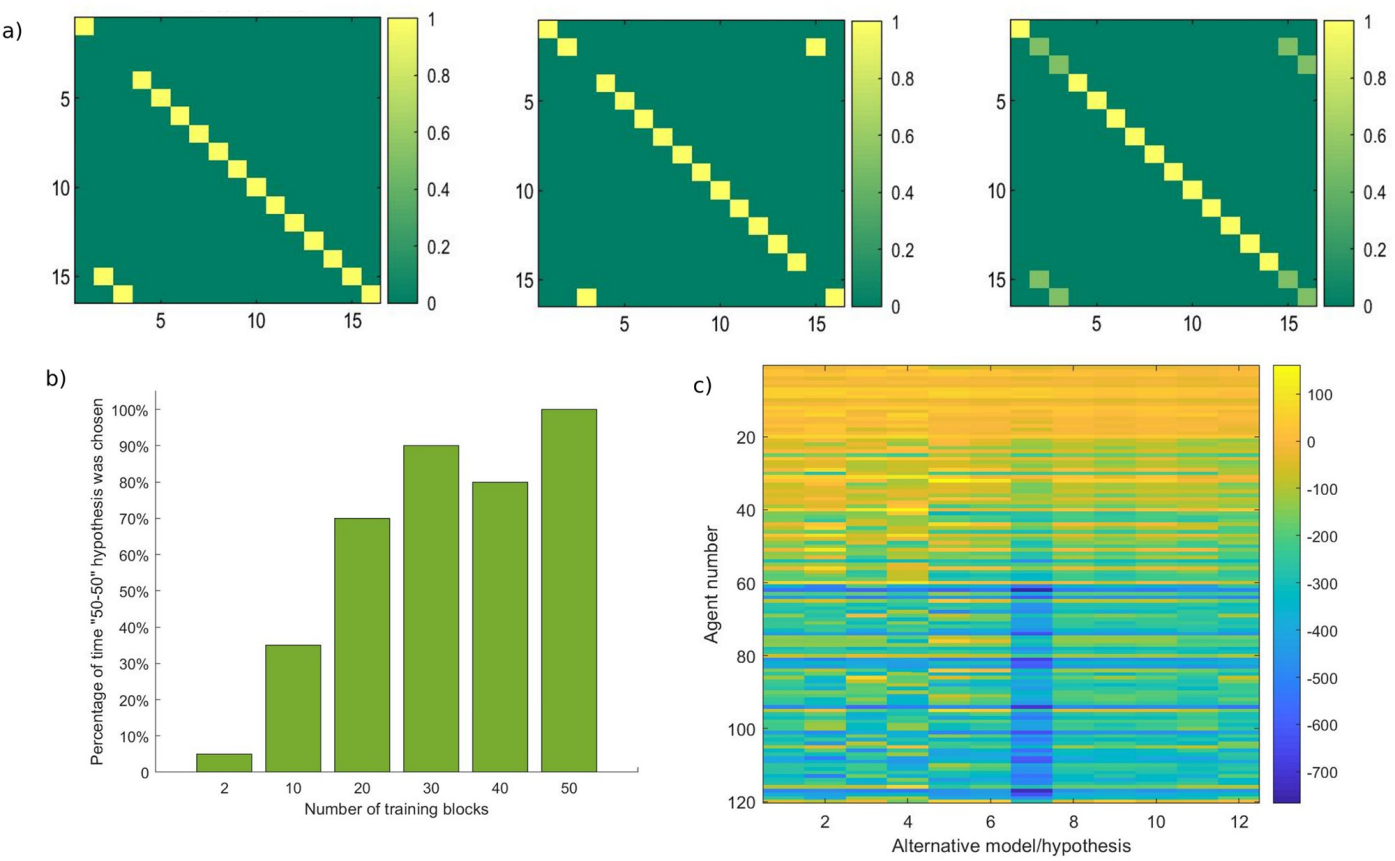
Possible representations of *similarity* between the rooms for three different agents after BMR and the most frequently chosen hypothesis during BMR. a) Likelihood matrices representing the reduced posterior concentration parameters. The matrices represent the *context* state-outcome mappings with rows representing the context state, and the columns representing the context outcome. The likelihood mapping for the first agent shows that rooms 2&15 as having the identity of context 15, and rooms 3&16 as being context 16. The second agent’s beliefs show that rooms 3&16 have the identity of context (room) 16, and rooms 2&15 as having the identity of context 2. The third agent believes that there is an equal probability for the rooms that are identical in terms of their configuration: 2&15 can be either context 2 or 15 and rooms 3&16 are equally likely to be either context (room) 3 or 16. b) The percentage of time the hypothesis with an equal (‘50–50’) probability for the rooms with identical configurations was chosen by the agents, for different numbers of training blocks. At N = 50 (i.e., after 50 higher level training blocks) this hypothesis is chosen 100% of time–that is, all 20 agents training for 50 higher level blocks, selected this hypothesis as being the most parsimonious, explaining the observations with the least model complexity. c) The negative log evidence for the twelve alternative hypotheses/models (x axis) for the entire set of agents (y axis, 120 agents). Model 7 appears to consistently have the greatest evidence (i.e., least free energy).

The agents come to recognise the rooms as being different only when their configurations could be disambiguated (i.e., as a result learning, rooms with different reward locations and contextual cue were not confused with each other). Fig 8a shows the final encoding of environmental structure (i.e., *context* mappings) for three different agents as examples. The agent on the left in Fig 8a believes that rooms 2&15 are room 15, and rooms 3&16 are room 16. The middle agent believes that rooms 2&15 are room 2, and rooms 3&16 are room 16. The agent on the right, however, believes that there is a 50% probability of being in either of the rooms with identical configurations. For example, when this agent is in room 15, it believes that it could be in either room 15 or room 2, with equal probability.

There is diversity in terms of these learnt mappings, based on variations in foraging the rooms as a result of pursuing different intrinsic and extrinsic affordances. As noted above, when implementing BMR for Dirichlet hyperparameters (in this case the *context* likelihood mappings), agents compute a relative log evidence (i.e., free energy) for each model, and compare this score to the evidence of the parent model. Subsequently, agents select the model with the greatest evidence (Fig 8b & 8c). The most frequently chosen alternative hypothesis (i.e., alternative model) is the one whereby there is a 50% probability of being in either of the rooms represented by identical configurations (i.e., model/hypothesis 7). Fig 8b also shows the percentage of time the alternative hypothesis (i.e., alternative model) with a 50–50 probability for the rooms with identical configurations was chosen when applying BMR for the entire set of agents (i.e., when applying BMR to all the agents after various training blocks), consisting of 120 synthetic agents.

**Fig 9:**
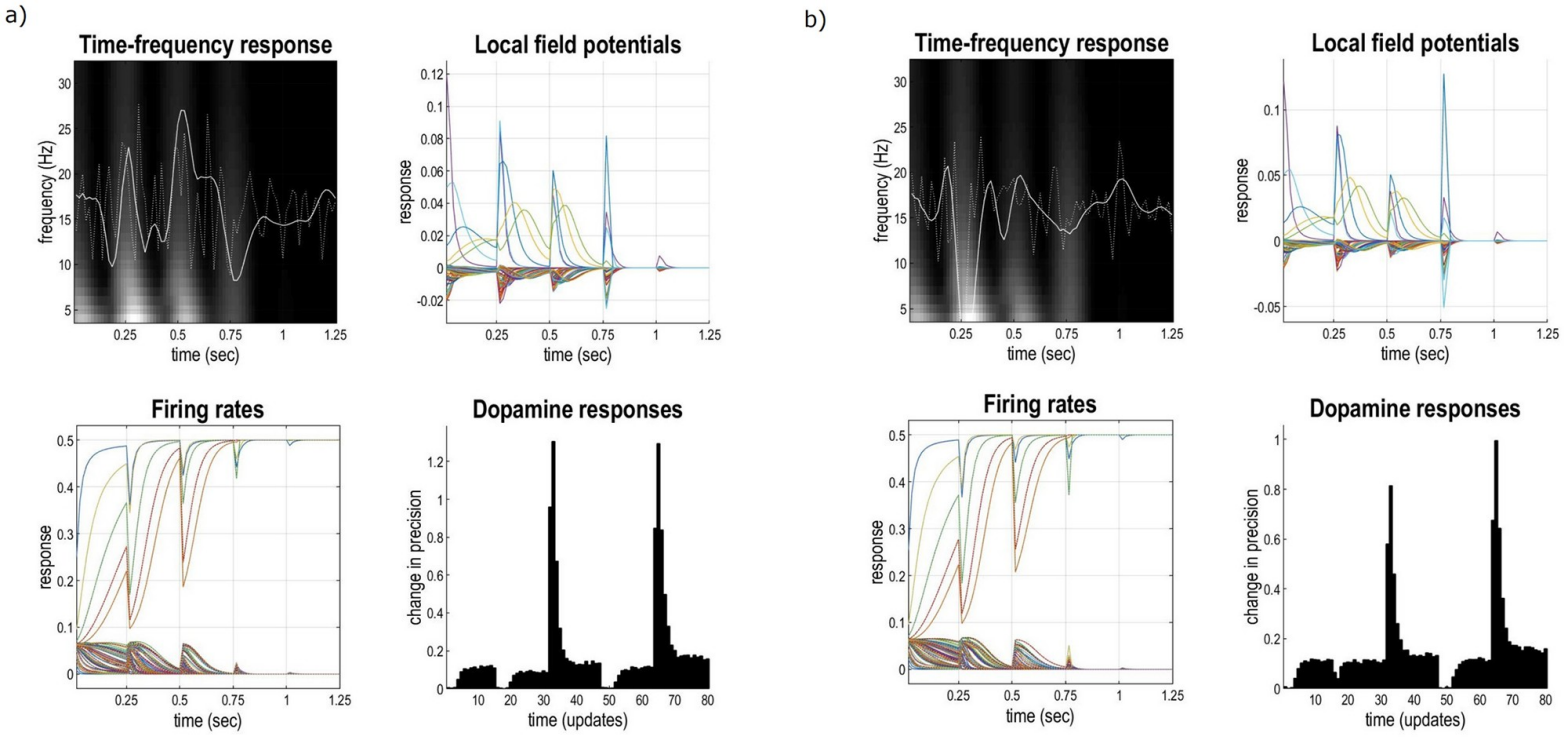
Neural activity for a synthetic agent in two rooms with identical configurations. In these epochs, the agent forages the two rooms in the same manner–that is, it follows the same trajectory of locations. During the last two steps, the agent encounters the reward and stays with the reward for one more step. Please see main text above for more details. a) Room 2 b) Room 15.

Interestingly, in addition to learning associations that encode similarity between rooms, in some cases agents also showed a similarity in simulated neural activity as characterised by (simulated) local field potentials, firing rates and dopaminergic responses. We illustrate the electrophysiological responses, associated with belief updating, for *one agent* foraging two rooms with identical configurations (rooms 2 and 15), during the training blocks (N = 50) (Fig 9). The agent follows a similar trajectory in these two rooms, gathering reward for the last two time-steps. The top-left panel of Fig 9 shows average local field potentials over all the units encoding the *context* factor before (dotted line) and after (solid line) bandpass filtering at 4Hz, juxtaposed with its time frequency decomposition. The lower-left panel illustrates evidence accumulation for these units. The top-right panel shows the rate of change of neuronal firing. Finally, the lower-right panel illustrates simulated dopaminergic responses defined as an amalgamation of precision and its rate of change. This is reminiscent of demonstrations (using Representational Similarity Analysis) that neural activity patterns to repeated presentations of identical or related stimuli are likewise very similar [41]. Please see the S1 Appendix for a contrast in electrophysiological activity that ensues as a result of *the same agent foraging the same room*, *with the same trajectory* (i.e., Room 15) at two different trials, and of *foraging different rooms* (i.e., Rooms 4 and 12) *with a similar trajectory*.

Posterior beliefs about policies are obtained by applying a softmax function to precision weighted (negative) expected free energy of each policy. The precision parameter is estimated as new observations become available, and it plays the role of an inverse temperature, meaning that the policy with the least expected free energy becomes more likely to be selected if the precision parameter is high. In other words, this precision encodes the confidence that the inferred policies will lead to preferred outcomes or resolves uncertainty about the hidden states. Previous work [16] suggests that the dopaminergic activity in the mid-brain might encode this kind of precision. In our paradigm, the phasic bursts we see in simulated dopaminergic responses indicate that at step 2 (i.e., the 32^nd^ iteration in terms of updates–Fig 9a and 9b, bottom right panels) the agent becomes more confident (i.e., resolves uncertainty) about which policies to pursue, having eliminated the possibility that the room it is foraging is room 14, given that it did not discover a reward at location 6, which is the rewarding location for room 14. During the second spike at step 4 (i.e., the 64^th^ iteration) the agent eliminates further possible policies, having become more confident that the room it is foraging is neither room 3 nor 16 (with reward at location 9), since it found a reward at location 1. This example illustrates how one can unpack belief updating and decision-making, while encoding uncertainty and precision.

### 4.4 The strength of prior preferences impacts concept formation

**Fig 10:**
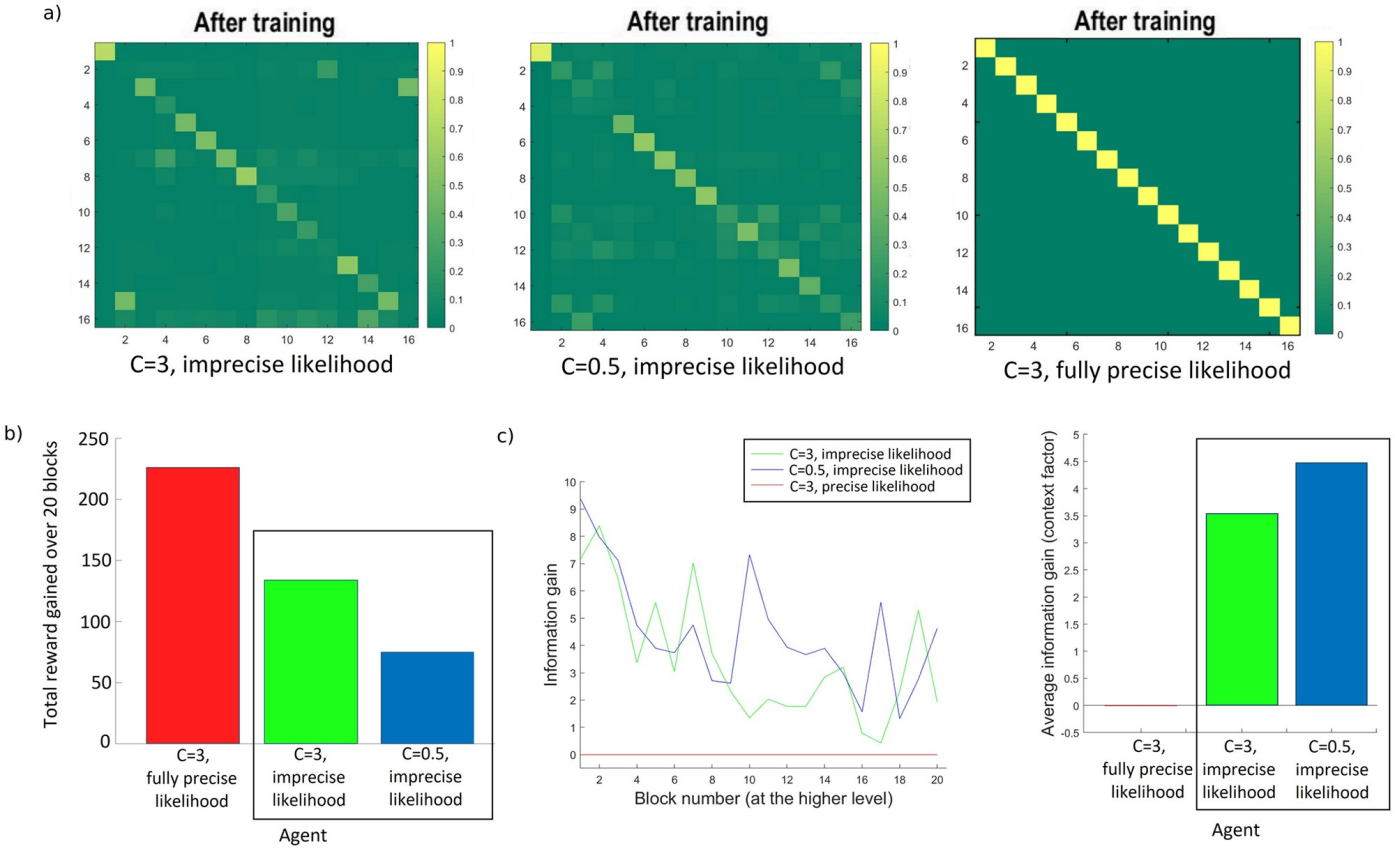
Performance comparison between an agent with a strong preference for reward (C = 3) versus an agent with a weaker preference (C = 0.5). a) Likelihood mappings after 20 training blocks, including a fully knowledgeable agent (right). b) Total reward accumulated over 20 (higher level) blocks. c) Comparison between the two agents, in terms of the information gain associated with the *context* modality.

One source of individual differences in concept formation reflects the preference for some outcomes over others. We asked whether prior preferences (i.e., regarding reward) influence learning and subsequent performance, by simulating three agents who experienced the same number of training blocks at the higher level (N = 20). For one agent, we reduced the precision of prior preference over outcomes (reward) to 0.5 and 0 elsewhere (as compared to the default used in all other simulations of 3 and 0 elsewhere). This means that the agent has a weaker reward preference, compared to its conspecifics. Fig 10a shows the learned *context* likelihood matrices for the agents that have different degrees of preferences for reward. The third agent (Fig 10a, right) starts with a fully precise set of likelihood matrices. We use this agent as a baseline to help illustrate the performance comparison between agents with higher and lower precision in prior preferences, providing a cap on the total amount of rewards that agents can gather. The agent with weaker preferences does not accumulate as much reward (Fig 10b), but learns more (Fig 10c). Here, the degree of learning was assessed with the information gain or KL divergence between posterior and prior Dirichlet concentration parameters for the *context* likelihood matrix. This quantifies how much the agent has learned about the state-outcome associations from the start of the simulations. This example shows that the agent with a weaker preference for rewards is more sensitive to epistemic incentives, and subsequently, learns more efficiently.

## 5. Discussion

This work focused on the importance of structure learning as implemented by Bayesian model reduction. We presented a series of simulations: we first established that agents form concepts and gather rewards as a result of interacting with their environment. Consequently we demonstrated the benefits of Bayesian model reduction, defined as an improvement in performance. Synthetic agents foraged a novel environment defined as a set of (16) rooms (contexts), each having 16 available locations. Agents started with an imprecise set of representations about the potential types of room (contexts)–in terms of possible reward locations–and a uniform distribution over the set of contexts in terms of their identity. That is, agents were unaware of which specific context they are in when they started foraging, but they had some representation about the possible configurations they could encounter. Without knowing the context they find themselves in, agents can only find the reward by chance. Agents came to learn the identities of the rooms (via updates to concentration parameters), and form precise beliefs about the structure of the environment they were foraging i.e., they formed concepts. By learning their environment, agents gradually start performing better–they develop the ability to find the reward more often, and gather more reward overall.

Bayesian model reduction enhanced the implicit concept acquisition and formation, endowing (synthetic) agents with representations that are more precise. The BMR process does not prune associations unless they combine high complexity with an inability to explain observations accurately. In sections 4.1 and 4.2 we saw that the parametric beliefs encoding the likelihood mappings of the *context* factor change (i.e., diverge from the initial uniform beliefs) depending upon the amount of training and whether or not BMR has been used. There was a marked difference in information gain after BMR relative to before BMR. Undergoing BMR entails higher information gain with regards to the parameters encoding the (state-outcome) representations. To our knowledge, this is the first attempt to compare information gain in Active Inference agents using BMR–by comparing between information gain following BMR, and information gain as a result of parametric learning. Future work could use this metric to ascertain how and when it would be most useful for both machines and humans to engage in structure learning.

Interestingly, during the learning (i.e., training) process, agents occasionally ‘mislabel’ rooms; i.e., appear to be confusing room identities. This is most likely due to the way these agents forage the environment: for example, if we have one agent foraging in a room, and the foraged locations do not contain the reward, the agent is likely to label this room as an other room whose corresponding locations do not contain a reward, everything else being equal.

Further to the effect of increased precision in concept formation, we have illustrated the performance benefits of BMR (section 4.2) in the context of goal-directed behaviour. In these numerical experiments, alternative hypotheses about the structure of the likelihood matrix (i.e., the agent’s beliefs about the set of rooms and their identities) were entertained. Selecting the most parsimonious hypothesis (i.e., the one with the greatest evidence) allowed the agents to minimise their uncertainty about their environment, and to use this knowledge (i.e., room identity as defined by its cue) to secure rewards. For example, after BMR, agents become more confident that the room they are foraging is pastel orange (i.e., room 6), and they can use this information to head to the reward (left and up on the first move), rather than responding to epistemic affordances or novelty. Agents undergoing BMR performed consistently better than agents not undergoing BMR. Most importantly, agents with BMR performed well, even before foraging all the rooms. This is important because it speaks to the ability of generative models with higher evidence to be generalised to new data and contexts [80]. This reflects the implicit idea behind [38,40,41] whereby the reorganisation and restructuring of information changes the form of concepts, regardless of whether more external information is assimilated or not. We have also seen this aspect in the problem-solving literature, where no further sensorial (or factual) information is necessary for insight [49,56–59]. Furthermore, Bayesian model reduction has been associated with physiological processes such as the regression of synaptic connections or pruning, observed during periods of sleep [63]. In this setting, the structure of generative models is learned by minimising model complexity in the absence of sensory data, when accuracy does not contribute to log evidence [81,82]. For example, in sleep, endogenous activity–that resembles neural message passing in wakefulness–has been interpreted as the generation of fictive data to evaluate model evidence: c.f., [83]. That is, fictive episodes are ‘replayed’, in the absence of (precise) sensory information, in order to optimise generative models (with the implication that this kind of model reduction facilitates generalisation).

Concept learning and formation were defined as a basic cognitive function, whereby (biological or artificial) agents identify and update beliefs about conditional dependencies and independencies in the environment. In problem solving, there is a slow, systematic process, and a fast process; namely, insight (defined as a sudden recognition of task-dependent contingencies). Although at first sight these processes appear to be disparate cognitive tools, they both appear to be underlined by the same computational mechanisms: Active Inference, parameter learning, and model selection. One might suggest that they have a similar teleology: to compare and organise how things relate to one another, thereby systematising relational knowledge to form parsimonious representations. Both these strategies imply a resolution of uncertainty about the causes of sensorial experience, behaviour, parameters of their model and the model *per se*. Biological agents can form and update representations in a gradual, systematic fashion, their learning punctuated with sudden realisations (i.e., resolution of uncertainty about the best model), regardless of what the representations themselves are about (e.g., the concept of a ‘living room’ or how to best arrange tiles in a bathroom). As we have seen in our simulations, agents can either learn the identities of the rooms gradually, or invoke BMR, and settle on a reduced model in order to continue foraging familiar rooms and collect familiar rewards.

Drawing parallels to the literature on insight [49,56–59], one can associate the number of training trials before invoking BMR as a ‘forced’ time-step threshold at which agents ‘reach’ an impasse. This moment precedes reorganisation of accumulated associations (i.e., model comparison), where different hypotheses about the configuration of rooms come into play. The ‘Aha’ moment can be thought of as the point at which the agent has ‘decided’ upon a particular hypothesis (i.e., model selection). Furthermore, we have seen in section 4.3 that agents can exhibit a pronounced inter-agent variability: a characteristic that pertains to the outcomes sampled, rather than the agents themselves. This has further implications in the realm of individual differences, because it can potentially elucidate how different individuals sampling different (sensorial) observations can reach the same conclusion (i.e., alternative hypothesis defining contingencies in the outside world), as well as how similar individuals sampling similar observations can reach different conclusions, as observed for example in [84], where individuals exposed to similar sensory information diverged in terms of whether they perceived a (bistable) stimulus as a vase or face. A possible limitation of this current work is the small number of alternative hypotheses (i.e., models). There is a combinatorial explosion for a 16x16 grid of possible room types given the contextual cue, resulting in a limited set of feasible alternative hypotheses (i.e., 12 in number). These arguments call for a better understanding of how the abstract alternative hypotheses compared for model selection are themselves generated, represented, and used both within and between agents. In other words, what are the rules that govern which hypotheses to select among–and are there models of models?

We have shown that as agents forage and learn about their environment, they also come to ascribe the same identity to rooms with similar configurations (i.e., colour and reward location). Furthermore, the similarity in representation was accompanied by very similar neurophysiological responses, as seen empirically in the concept learning literature by Love and Gureckis [38], Love, Medin [39]. It remains to be seen when this is or is not the case across the board. For example, whether similar representations at time-points far apart evoke this same effect, or whether there is a relationship describing discrepancies between items of the same class. Interestingly, in sections 4.1 and 4.3, we saw that in the first instance (i.e., N = 2, N = 10), Bayesian model reduction appears to promote generalisation, with agents perceiving the rooms with identical configurations as being one room. After more experience however, the agents’ beliefs seem to diverge again, ‘perceiving’ the rooms with similar configurations as having a 50–50% probability of being each of the two possible rooms. That is to say that agents are retaining both representations in an attempt to maintain a ‘flexible’ set of beliefs, regardless of having evidence to the contrary (i.e., that they do not need two separate concepts). A future research direction could identify this potential computational benefit of developing and retaining a ‘flexible’ set of beliefs about contingencies in the lived world, in light of the Active Inference account. That is, what are the useful measures when deciding whether to retain a more flexible but less precise set of beliefs, versus a more rigid but also more precise set of beliefs?

Finally, we considered one source of individual differences, namely the strength of prior preferences for reward. Preferences affected concept acquisition and therefore the way agents formed representations. An agent with an imprecise preference for reward explored its environment more, and diverged more from its prior beliefs encoding state-outcome associations. Making more exploratory choices in this case hindered performance, in terms of reward gained, as well as the number of times the reward was found. These results are reminiscent of work by [85]: here, the authors demonstrate that in Active Inference, uncertainties pertaining to the agents’ goals and preferences are prioritised over other types of uncertainty. Active inference thus provides a Bayes optimal and principled approach to balancing epistemic (i.e., exploratory) and instrumental (i.e., exploitative) actions. As predicted by [85], this balance depends on the shape of agents’ beliefs; in our case underwritten by prior preferences. In light of these (and our results), agents minimise uncertainty insofar as it is required for fulfilling their goals, whatever they may be defined as. When the imperative for satisfying prior preferences is diminished in relation to epistemic imperatives, the balance between exploration and exploitation shifts towards explorative behaviour, and vice versa. Future computational and empirical work may involve assessing agents with different levels of reward preferences, to see whether agents with a strong preference end up forfeiting exploratory behaviour (and, with it, predictive power) in an attempt to obtain rewards.

Structure learning as implemented by Bayesian model selection (here, Bayesian model reduction) is an emerging topic in the computational neurosciences. The literature on this burgeoning topic is currently limited. Part of the motivation for this work was to expand on this literature and foreground the importance of this type of learning (i.e., learning in the absence of new evidence), and how it can influence belief formation and updating. In summary, we have presented a deep hierarchical Active Inference model of goal directed behaviour, and the associated optimisation schemes implied by maximising model evidence. We have used numerical experiments to showcase a series of potential mechanisms that underwrite concept learning in a spatial foraging task. Synthetic agents formed concepts about the identities and configurations of the ‘rooms’ in a synthetic environment as a result of free energy minimising inference, learning, and model selection processes–three processes that contextualise each other and can produce a diversity of beliefs and belief structures. Structure learning as implemented by Bayesian model reduction enhanced concept formation, showing an improvement in performance as scored by information gain and the amount of reward gathered. Furthermore, the representations formed as a result of these three processes reflected symmetries for ‘rooms’ with identical configurations (i.e., reward location and contextual cue). Finally, we have shown that the shape of prior beliefs (in this case, the strength of prior preferences for reward) affects the balance between exploration and exploitation.

### Software note

Although the generative model specified by the (A, B, C, and D) matrices—changes from application to application, the belief updates in Figs 1 and 3 are generic and can be implemented using standard routines (spm_MDP_VB_X.m). These routines are available as MATLAB code in the SPM academic software: http://www.fil.ion.ucl.ac.uk/spm/.

## Supporting information

S1 Appendix Neural activity comparison for four further rooms. Panels a) and b) in the figure show Room 15 with the agent adopting the same trajectory (locations 7, 11, 10, 14, 14) at 2 different instances: a) block 28 and b) block 30. Neural activity appears to be similar, as anticipated. Panels c) and d) compare different rooms with the same trajectory (locations 7, 11, 10, 14, and 14), who’s neurophysiological activity also differs in spite of having a similar trajectory. Panel c) depicts simulated electrophysiological activity for Room 4 and panel d) shows activity for Room 12.
(TIF)

## References

[1] Da Costa L, Parr T, Sajid N, Veselic S, Neacsu V, Friston K. Active inference on discrete state-spaces: A synthesis. Journal of Mathematical Psychology. 2020;99:102447. doi: 10.1016/j.jmp.2020.102447 33343039 PMC7732703

[2] Friston K, Buzsáki G. The Functional Anatomy of Time: What and When in the Brain. Trends Cogn Sci. 2016;20(7):500–11. doi: 10.1016/j.tics.2016.05.001 27261057

[3] Friston K, FitzGerald T, Rigoli F, Schwartenbeck P, O’Doherty J, Pezzulo G. Active inference and learning. Neuroscience & Biobehavioral Reviews. 2016;68:862–79. 27375276 10.1016/j.neubiorev.2016.06.022 PMC5167251

[4] Friston K, FitzGerald T, Rigoli F, Schwartenbeck P, Pezzulo G. Active Inference: A Process Theory. Neural Computation. 2017;29(1):1–49. doi: 10.1162/NECO_a_00912 27870614

[5] Friston K, Schwartenbeck P, Fitzgerald T, Moutoussis M, Behrens T, Dolan R. The anatomy of choice: active inference and agency. Front Hum Neurosci. 2013;7(598). doi: 10.3389/fnhum.2013.00598 24093015 PMC3782702

[6] Friston K, Schwartenbeck P, FitzGerald T, Moutoussis M, Behrens T, Dolan RJ. The anatomy of choice: dopamine and decision-making. Philosophical Transactions of the Royal Society B: Biological Sciences. 2014;369(1655):20130481. doi: 10.1098/rstb.2013.0481 25267823 PMC4186234

[7] Friston KJ, Parr T, de Vries B. The graphical brain: Belief propagation and active inference. Netw Neurosci. 2017;1(4):381–414. doi: 10.1162/NETN_a_00018 29417960 PMC5798592

[8] Friston KJ, Rosch R, Parr T, Price C, Bowman H. Deep temporal models and active inference. Neuroscience & Biobehavioral Reviews. 2017;77:388–402. doi: 10.1016/j.neubiorev.2017.04.009 28416414 PMC5461873

[9] Hesp C, Smith R, Parr T, Allen M, Friston KJ, Ramstead MJD. Deeply Felt Affect: The Emergence of Valence in Deep Active Inference. Neural Comput. 2021;33(2):398–446. doi: 10.1162/neco_a_01341 33253028 PMC8594962

[10] Kaplan R, Friston KJ. Planning and navigation as active inference. Biological Cybernetics. 2018;112(4):323–43. doi: 10.1007/s00422-018-0753-2 29572721 PMC6060791

[11] Mirza MB, Adams RA, Friston K, Parr T. Introducing a Bayesian model of selective attention based on active inference. Scientific Reports. 2019;9(1):13915. doi: 10.1038/s41598-019-50138-8 31558746 PMC6763492

[12] Mirza MB, Adams RA, Mathys CD, Friston KJ. Scene Construction, Visual Foraging, and Active Inference. Frontiers in computational neuroscience. 2016;10:56-. doi: 10.3389/fncom.2016.00056 27378899 PMC4906014

[13] Neacsu V, Convertino L, Friston KJ. Synthetic Spatial Foraging With Active Inference in a Geocaching Task. Frontiers in Neuroscience. 2022;16. 10.3389/fnins.2022.802396 PMC8861269 35210988

[14] Parr T, Friston KJ. Uncertainty, epistemics and active inference. Journal of the Royal Society Interface. 2017;14(136):20170376. doi: 10.1098/rsif.2017.0376 29167370 PMC5721148

[15] Parr T, Friston KJ. Active inference and the anatomy of oculomotion. Neuropsychologia. 2018;111:334–43. doi: 10.1016/j.neuropsychologia.2018.01.041 29407941 PMC5884328

[16] Schwartenbeck P, FitzGerald TH, Mathys C, Dolan R, Friston K. The Dopaminergic Midbrain Encodes the Expected Certainty about Desired Outcomes. Cereb Cortex. 2015;25(10):3434–45. doi: 10.1093/cercor/bhu159 25056572 PMC4585497

[17] Seth AK, Friston KJ. Active interoceptive inference and the emotional brain. Philosophical Transactions of the Royal Society B: Biological Sciences. 2016;371(1708):20160007. doi: 10.1098/rstb.2016.0007 28080966 PMC5062097

[18] Friston KJ, Parr T, Zeidman P. Bayesian model reduction. arXiv: Methodology. 2018.

[19] Smith R, Schwartenbeck P, Parr T, Friston KJ. An Active Inference Approach to Modeling Structure Learning: Concept Learning as an Example Case. Frontiers in Computational Neuroscience. 2020;14:41. doi: 10.3389/fncom.2020.00041 32508611 PMC7250191

[20] Bruner JS, Goodnow JJ, Austin GA. A study of thinking. Oxford, England: John Wiley and Sons; 1956. xi, 330–xi, p.

[21] Geeraerts D. Prototype theory. Cognitive linguistics: Basic readings. 2006;34:141–65.

[22] Goodman ND, Tenenbaum JB, Feldman J, Griffiths TL. A rational analysis of rule-based concept learning. Cogn Sci. 2008;32(1):108–54. doi: 10.1080/03640210701802071 21635333

[23] Rouder JN, Ratcliff R. Comparing categorization models. J Exp Psychol Gen. 2004;133(1):63–82. doi: 10.1037/0096-3445.133.1.63 14979752 PMC1403834

[24] Barsalou LW. Ad hoc categories. Memory & Cognition. 1983;11(3):211–27. doi: 10.3758/bf03196968 6621337

[25] Blei DM, Griffiths TL, Jordan MI, Tenenbaum JB, editors. Hierarchical topic models and the nested Chinese restaurant process. NIPS; 2003.

[26] Gershman SJ, Blei DM. A tutorial on Bayesian nonparametric models. Journal of Mathematical Psychology. 2012;56(1):1–12.

[27] Griffiths TL, Sanborn AN, Canini KR, Navarro DJ, Tenenbaum JB. Nonparametric Bayesian models of categorization. Formal approaches in categorization. 2011:173–98.

[28] Stoianov I, Genovesio A, Pezzulo G. Prefrontal goal codes emerge as latent states in probabilistic value learning. Journal of Cognitive Neuroscience. 2016;28(1):140–57. doi: 10.1162/jocn_a_00886 26439267

[29] Collins AG, Frank MJ. Cognitive control over learning: creating, clustering, and generalizing task-set structure. Psychological review. 2013;120(1):190. doi: 10.1037/a0030852 23356780 PMC3974273

[30] McNicholas PD. Model-based clustering. Journal of Classification. 2016;33(3):331–73.

[31] Salakhutdinov R, Tenenbaum JB, Torralba A. Learning with hierarchical-deep models. IEEE transactions on pattern analysis and machine intelligence. 2012;35(8):1958–71. 10.1109/TPAMI.2012.269 23787346

[32] Mnih V, Kavukcuoglu K, Silver D, Rusu AA, Veness J, Bellemare MG, et al. Human-level control through deep reinforcement learning. nature. 2015;518(7540):529–33. doi: 10.1038/nature14236 25719670

[33] Fourment M, Magee AF, Whidden C, Bilge A, Matsen FA IV, Minin VN. 19 dubious ways to compute the marginal likelihood of a phylogenetic tree topology. Systematic biology. 2020;69(2):209–20. doi: 10.1093/sysbio/syz046 31504998 PMC7571498

[34] Penny WD. Comparing dynamic causal models using AIC, BIC and free energy. Neuroimage. 2012;59(1):319–30. doi: 10.1016/j.neuroimage.2011.07.039 21864690 PMC3200437

[35] Barron HC, Auksztulewicz R, Friston K. Prediction and memory: A predictive coding account. Prog Neurobiol. 2020;192:101821. doi: 10.1016/j.pneurobio.2020.101821 32446883 PMC7305946

[36] Bowman CR, Zeithamova D. Abstract Memory Representations in the Ventromedial Prefrontal Cortex and Hippocampus Support Concept Generalization. The Journal of Neuroscience. 2018;38(10):2605–14. doi: 10.1523/JNEUROSCI.2811-17.2018 29437891 PMC5858598

[37] Hutter SA, Wilson AI. A Novel Role for the Hippocampus in Category Learning. The Journal of neuroscience: the official journal of the Society for Neuroscience. 2018;38(31):6803–5. doi: 10.1523/JNEUROSCI.1085-18.2018 30995610 PMC6596119

[38] Love BC, Gureckis TM. Models in search of a brain. Cogn Affect Behav Neurosci. 2007;7(2):90–108. doi: 10.3758/cabn.7.2.90 17672381

[39] Love BC, Medin DL, Gureckis TM. SUSTAIN: a network model of category learning. Psychol Rev. 2004;111(2):309–32. doi: 10.1037/0033-295X.111.2.309 15065912

[40] Mack ML, Love BC, Preston AR. Building concepts one episode at a time: The hippocampus and concept formation. Neurosci Lett. 2018;680:31–8. doi: 10.1016/j.neulet.2017.07.061 28801273 PMC5803467

[41] Mack ML, Love BC, Preston AR. Dynamic updating of hippocampal object representations reflects new conceptual knowledge. Proceedings of the National Academy of Sciences. 2016;113(46):13203–8. doi: 10.1073/pnas.1614048113 27803320 PMC5135299

[42] Mack ML, Preston AR, Love BC. Ventromedial prefrontal cortex compression during concept learning. Nature Communications. 2020;11(1):46. doi: 10.1038/s41467-019-13930-8 31911628 PMC6946809

[43] McClelland JL, McNaughton BL, O’Reilly RC. Why there are complementary learning systems in the hippocampus and neocortex: insights from the successes and failures of connectionist models of learning and memory. Psychol Rev. 1995;102(3):419–57. doi: 10.1037/0033-295X.102.3.419 7624455

[44] Mok RM, Love BC. A non-spatial account of place and grid cells based on clustering models of concept learning. Nature Communications. 2019;10(1):5685. doi: 10.1038/s41467-019-13760-8 31831749 PMC6908717

[45] Zeithamova D, Mack ML, Braunlich K, Davis T, Seger CA, van Kesteren MTR, et al. Brain Mechanisms of Concept Learning. J Neurosci. 2019;39(42):8259–66. doi: 10.1523/JNEUROSCI.1166-19.2019 31619495 PMC6794919

[46] Eichenbaum H. Prefrontal–hippocampal interactions in episodic memory. Nature Reviews Neuroscience. 2017;18(9):547–58. doi: 10.1038/nrn.2017.74 28655882

[47] Gruber MJ, Hsieh L-T, Staresina BP, Elger CE, Fell J, Axmacher N, et al. Theta phase synchronization between the human hippocampus and prefrontal cortex increases during encoding of unexpected information: a case study. Journal of Cognitive Neuroscience. 2018;30(11):1646–56. doi: 10.1162/jocn_a_01302 29952700

[48] Rubin RD, Schwarb H, Lucas HD, Dulas MR, Cohen NJ. Dynamic hippocampal and prefrontal contributions to memory processes and representations blur the boundaries of traditional cognitive domains. Brain sciences. 2017;7(7):82. doi: 10.3390/brainsci7070082 28704928 PMC5532595

[49] Weisberg RW. On the “Demystification” of Insight: A Critique of Neuroimaging Studies of Insight. Creativity Research Journal. 2013;25(1):1–14.

[50] Knoblich G, Ohlsson S, Raney GE. An eye movement study of insight problem solving. Mem Cognit. 2001;29(7):1000–9. doi: 10.3758/bf03195762 11820744

[51] MacGregor JN, Ormerod TC, Chronicle EP. Information processing and insight: a process model of performance on the nine-dot and related problems. Journal of experimental psychology Learning, memory, and cognition. 2001;27(1):176–201. 11204097

[52] Jones G. Testing two cognitive theories of insight. Journal of experimental psychology Learning, memory, and cognition. 2003;29(5):1017–27. doi: 10.1037/0278-7393.29.5.1017 14516232

[53] Mai XQ, Luo J, Wu JH, Luo YJ. "Aha!" effects in a guessing riddle task: an event-related potential study. Hum Brain Mapp. 2004;22(4):261–70. doi: 10.1002/hbm.20030 15202104 PMC6871977

[54] Bowden EM, Jung-Beeman M, Fleck J, Kounios J. New approaches to demystifying insight. Trends Cogn Sci. 2005;9(7):322–8. doi: 10.1016/j.tics.2005.05.012 15953756

[55] Friston KJ, Lin M, Frith CD, Pezzulo G, Hobson JA, Ondobaka S. Active Inference, Curiosity and Insight. Neural Computation. 2017;29(10):2633–83. doi: 10.1162/neco_a_00999 28777724

[56] Kounios J, Fleck JI, Green DL, Payne L, Stevenson JL, Bowden EM, et al. The origins of insight in resting-state brain activity. Neuropsychologia. 2008;46(1):281–91. doi: 10.1016/j.neuropsychologia.2007.07.013 17765273 PMC2293274

[57] Kounios J, Frymiare JL, Bowden EM, Fleck JI, Subramaniam K, Parrish TB, et al. The Prepared Mind:Neural Activity Prior to Problem Presentation Predicts Subsequent Solution by Sudden Insight. Psychological Science. 2006;17(10):882–90. doi: 10.1111/j.1467-9280.2006.01798.x 17100789

[58] Shen W, Tong Y, Li F, Yuan Y, Hommel B, Liu C, et al. Tracking the neurodynamics of insight: A meta-analysis of neuroimaging studies. Biological Psychology. 2018;138:189–98. doi: 10.1016/j.biopsycho.2018.08.018 30165082

[59] Tik M, Sladky R, Luft CDB, Willinger D, Hoffmann A, Banissy MJ, et al. Ultra-high-field fMRI insights on insight: Neural correlates of the Aha!-moment. Human Brain Mapping. 2018;39(8):3241–52. doi: 10.1002/hbm.24073 29665228 PMC6055807

[60] Luo J, Niki K. Function of hippocampus in “insight” of problem solving. Hippocampus. 2003;13(3):316–23. doi: 10.1002/hipo.10069 12722972

[61] Adams RA, Moutoussis M, Nour MM, Dahoun T, Lewis D, Illingworth B, et al. Variability in Action Selection Relates to Striatal Dopamine 2/3 Receptor Availability in Humans: A PET Neuroimaging Study Using Reinforcement Learning and Active Inference Models. Cerebral Cortex. 2020;30(6):3573–89. doi: 10.1093/cercor/bhz327 32083297 PMC7233027

[62] Brown TH, Zhao Y, Leung V. Hebbian Plasticity. In: Squire LR, editor. Encyclopedia of Neuroscience. Oxford: Academic Press; 2009. p. 1049–56.

[63] Tononi G, Cirelli C. Sleep function and synaptic homeostasis. Sleep Med Rev. 2006;10(1):49–62. doi: 10.1016/j.smrv.2005.05.002 16376591

[64] Toutounji H, Pipa G. Spatiotemporal computations of an excitable and plastic brain: neuronal plasticity leads to noise-robust and noise-constructive computations. PLoS Comput Biol. 2014;10(3):e1003512. doi: 10.1371/journal.pcbi.1003512 24651447 PMC3961183

[65] Hohwy J. The Self-Evidencing Brain. Noûs. 2016;50(2):259–85.

[66] Constantino SM, Daw ND. Learning the opportunity cost of time in a patch-foraging task. Cogn Affect Behav Neurosci. 2015;15(4):837–53. doi: 10.3758/s13415-015-0350-y 25917000 PMC4624618

[67] Constant A, Ramstead MJD, Veissière SPL, Friston K. Regimes of Expectations: An Active Inference Model of Social Conformity and Human Decision Making. Frontiers in Psychology. 2019;10(679). doi: 10.3389/fpsyg.2019.00679 30988668 PMC6452780

[68] Smith R, Parr T, Friston KJ. Simulating emotions: An active inference model of emotional state inference and emotion concept learning. Frontiers in psychology. 2019;10:2844. doi: 10.3389/fpsyg.2019.02844 31920873 PMC6931387

[69] Winn J, Bishop CM. Variational message passing. Journal of Machine Learning Research. 2005;6:661–94.

[70] Friston K, Penny W. Post hoc Bayesian model selection. Neuroimage. 2011;56(4):2089–99. doi: 10.1016/j.neuroimage.2011.03.062 21459150 PMC3112494

[71] George D, Hawkins J. Towards a mathematical theory of cortical micro-circuits. PLoS Comput Biol. 2009;5(10):e1000532. doi: 10.1371/journal.pcbi.1000532 19816557 PMC2749218

[72] Haxby JV, Grady CL, Horwitz B, Ungerleider LG, Mishkin M, Carson RE, et al. Dissociation of object and spatial visual processing pathways in human extrastriate cortex. Proceedings of the National Academy of Sciences. 1991;88(5):1621–5. doi: 10.1073/pnas.88.5.1621 2000370 PMC51076

[73] Haxby JV, Horwitz B, Ungerleider LG, Maisog JM, Pietrini P, Grady CL. The functional organization of human extrastriate cortex: a PET-rCBF study of selective attention to faces and locations. Journal of neuroscience. 1994;14(11):6336–53. doi: 10.1523/JNEUROSCI.14-11-06336.1994 7965040 PMC6577268

[74] Spiegler BJ, Mishkin M. Evidence for the sequential participation of inferior temporal cortex and amygdala in the acquisition of stimulus-reward associations. Behavioural Brain Research. 1981;3(3):303–17. doi: 10.1016/0166-4328(81)90002-4 7306385

[75] Miller JF, Neufang M, Solway A, Brandt A, Trippel M, Mader I, et al. Neural activity in human hippocampal formation reveals the spatial context of retrieved memories. Science. 2013;342(6162):1111–4. doi: 10.1126/science.1244056 24288336 PMC4669102

[76] Rudy JW, Barrientos RM, O’Reilly RC. Hippocampal formation supports conditioning to memory of a context. Behavioral Neuroscience. 2002;116(4):530–8. doi: 10.1037//0735-7044.116.4.530 12148921

[77] Andersen RA. Encoding of intention and spatial location in the posterior parietal cortex. Cerebral Cortex. 1995;5(5):457–69. doi: 10.1093/cercor/5.5.457 8547792

[78] Colby CL, Duhamel J-R. Spatial representations for action in parietal cortex. Cognitive Brain Research. 1996;5(1–2):105–15. doi: 10.1016/s0926-6410(96)00046-8 9049076

[79] Silver MA, Kastner S. Topographic maps in human frontal and parietal cortex. Trends in cognitive sciences. 2009;13(11):488–95. doi: 10.1016/j.tics.2009.08.005 19758835 PMC2767426

[80] MacKay DJC. Information Theory, Inference and Learning Algorithms. Cambridge: Cambridge University Press; 2003.

[81] Hobson JA, Friston KJ. Waking and dreaming consciousness: neurobiological and functional considerations. Prog Neurobiol. 2012;98(1):82–98. doi: 10.1016/j.pneurobio.2012.05.003 22609044 PMC3389346

[82] Pezzulo G, Zorzi M, Corbetta M. The secret life of predictive brains: what’s spontaneous activity for? Trends in Cognitive Sciences. 2021. doi: 10.1016/j.tics.2021.05.007 34144895 PMC8363551

[83] Hinton GE, Dayan P, Frey BJ, Neal RM. The" wake-sleep" algorithm for unsupervised neural networks. Science. 1995;268(5214):1158–61. doi: 10.1126/science.7761831 7761831

[84] Finlayson NJ, Neacsu V, Schwarzkopf DS. Spatial heterogeneity in bistable figure-ground perception. i-Perception. 2020;11(5):2041669520961120. doi: 10.1177/2041669520961120 33194167 PMC7594238

[85] Tschantz A, Seth AK, Buckley CL. Learning action-oriented models through active inference. PLoS computational biology. 2020;16(4):e1007805. doi: 10.1371/journal.pcbi.1007805 32324758 PMC7200021

