# Supplementary figures and images for: Structure learning enhances concept formation in synthetic Active Inference agents
Source: PLoS One. 2022 Nov 14;17(11):e0277199. doi: 10.1371/journal.pone.0277199 (PMC9662737; doi:10.1371/journal.pone.0277199)

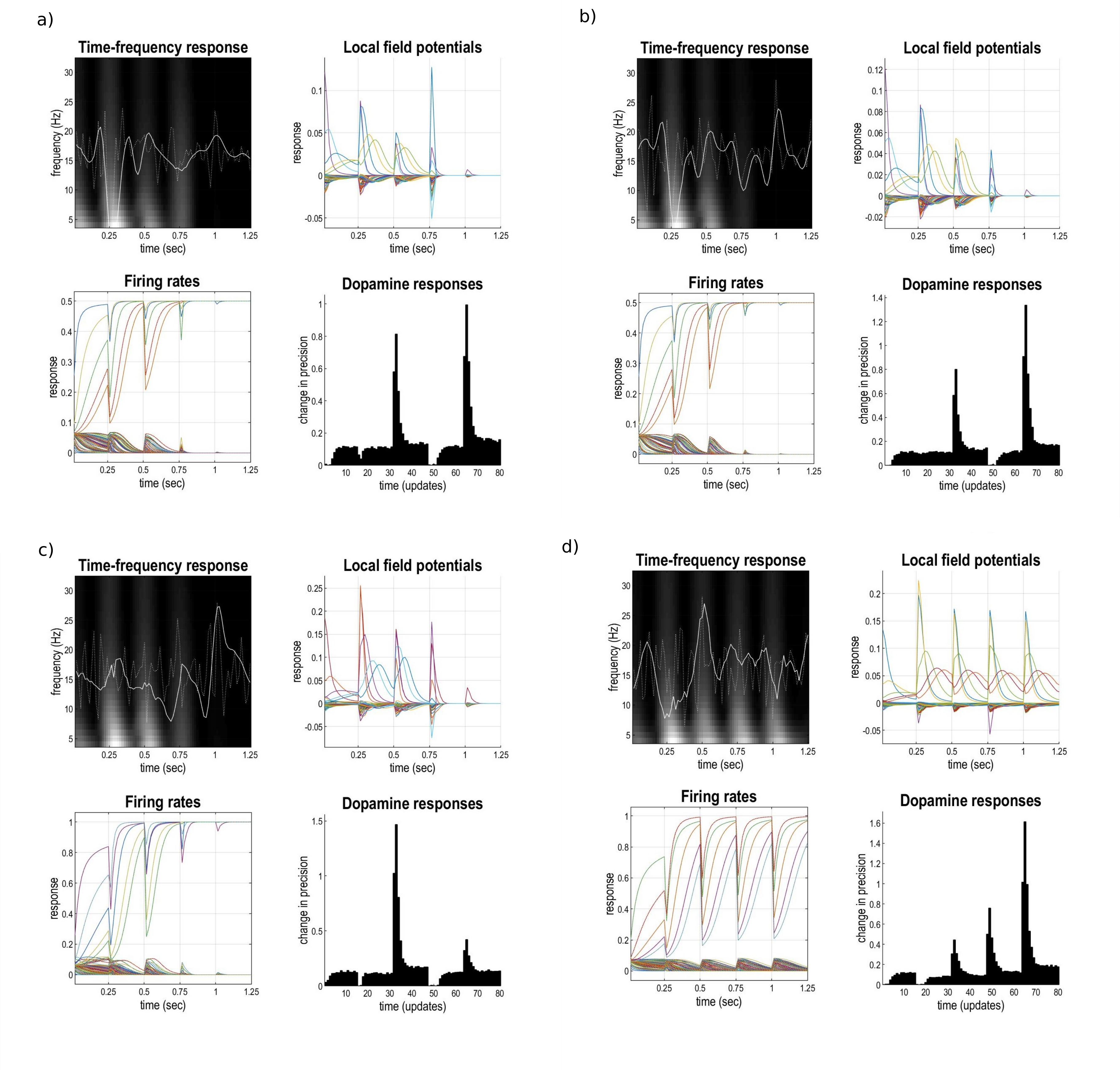

Supplement: S1 Appendix — Panels a) and b) in the figure show Room 15 with the agent adopting the same trajectory (locations 7, 11, 10, 14, 14) at 2 different instances: a) block 28 and b) block 30. Neural activity appears to be similar, as anticipated. Panels c) and d) compare different rooms with the same trajectory (locations 7, 11, 10, 14, and 14), who’s neurophysiological activity also differs in spite of having a similar trajectory. Panel c) depicts simulated electrophysiological activity for Room 4 and panel d) shows activity for Room 12. (TIF) [file pone.0277199.s001.tif]
